# Polycarcin V induces DNA-damage response and enables the profiling of DNA-binding proteins

**DOI:** 10.1093/nsr/nwac046

**Published:** 2022-03-11

**Authors:** Zongwei Yue, Fan Wu, Fusheng Guo, Jiyeong Park, Jin Wang, Liyun Zhang, Daohong Liao, Wenyang Li, Orlando D Schärer, Xiaoguang Lei

**Affiliations:** Beijing National Laboratory for Molecular Sciences, Key Laboratory of Bioorganic Chemistry and Molecular Engineering of Ministry of Education, Department of Chemical Biology, College of Chemistry and Molecular Engineering, Synthetic and Functional Biomolecules Center, Peking University, Beijing 100871, China; Beijing National Laboratory for Molecular Sciences, Key Laboratory of Bioorganic Chemistry and Molecular Engineering of Ministry of Education, Department of Chemical Biology, College of Chemistry and Molecular Engineering, Synthetic and Functional Biomolecules Center, Peking University, Beijing 100871, China; Beijing National Laboratory for Molecular Sciences, Key Laboratory of Bioorganic Chemistry and Molecular Engineering of Ministry of Education, Department of Chemical Biology, College of Chemistry and Molecular Engineering, Synthetic and Functional Biomolecules Center, Peking University, Beijing 100871, China; Peking-Tsinghua Center for Life Science, Academy for Advanced Interdisciplinary Studies, Peking University, Beijing 100871, China; Center for Genomic Integrity, Institute for Basic Science, Ulsan 44919, Republic of Korea; Beijing National Laboratory for Molecular Sciences, Key Laboratory of Bioorganic Chemistry and Molecular Engineering of Ministry of Education, Department of Chemical Biology, College of Chemistry and Molecular Engineering, Synthetic and Functional Biomolecules Center, Peking University, Beijing 100871, China; Beijing National Laboratory for Molecular Sciences, Key Laboratory of Bioorganic Chemistry and Molecular Engineering of Ministry of Education, Department of Chemical Biology, College of Chemistry and Molecular Engineering, Synthetic and Functional Biomolecules Center, Peking University, Beijing 100871, China; Jiangsu JITRI Molecular Engineering Inst. Co., Ltd., Suzhou 215500, China; Beijing National Laboratory for Molecular Sciences, Key Laboratory of Bioorganic Chemistry and Molecular Engineering of Ministry of Education, Department of Chemical Biology, College of Chemistry and Molecular Engineering, Synthetic and Functional Biomolecules Center, Peking University, Beijing 100871, China; Center for Genomic Integrity, Institute for Basic Science, Ulsan 44919, Republic of Korea; Department of Biological Sciences, School of Life Sciences, Ulsan National Institute of Science and Technology, Ulsan 44919, Republic of Korea; Beijing National Laboratory for Molecular Sciences, Key Laboratory of Bioorganic Chemistry and Molecular Engineering of Ministry of Education, Department of Chemical Biology, College of Chemistry and Molecular Engineering, Synthetic and Functional Biomolecules Center, Peking University, Beijing 100871, China; Peking-Tsinghua Center for Life Science, Academy for Advanced Interdisciplinary Studies, Peking University, Beijing 100871, China; Institute for Cancer Research, Shenzhen Bay Laboratory, Shenzhen 518107, China

**Keywords:** polycarcin V, [2+2] DNA photoadduct, light-activatable chemical probe, ABPP for DNA-binding protein proteomics, psoriasis treatment

## Abstract

To maintain genomic integrity and avoid diseases, the DNA-damage response (DDR) not only detects and repairs DNA lesions, but also contributes to the resistance to DNA-damaging chemotherapeutics. Targeting the DDR plays a significant role in drug discovery using the principle of synthetic lethality. The incomplete current knowledge of the DDR encouraged us to develop new strategies to identify and study its components and pathways. Polycarcin V, belonging to the C-aryl glycoside natural products, is a light-activatable DNA-intercalating agent that causes DNA damage by forming a covalent [2+2] cycloadduct with thymine residue under 365–450 nm of light irradiation in a DNA-sequence-independent manner. Taking advantage of the light-activatable feature and temporal control of DDR, we designed and synthesized polycarcin V-based bifunctional chemical probes, including one that cross-links DNA to DNA-binding protein to explore the DDR induced by polycarcin V and uncover novel DNA–protein interactions. Utilizing this chemical probe and activity-based protein profiling-stable isotope labeling with amino acids in cell culture, we identified 311 DNA-binding protein candidates, including known DDR factors and additional proteins that may be of interest in discovering new biology. We validated our approach by showing that our probe could specifically cross-link proteins involved in nucleotide excision repair (NER) that repair bulky DNA adducts. Our studies showed that the [2+2] cycloadduct formed by polycarcin V could indeed be repaired by NER *in vivo*. As a DNA-damaging agent, polycarcin V or its drug-like derivative plus blue light showed promising properties for psoriasis treatment, suggesting that it may itself hold promise for clinic applications.

## Teaser

Polycarcin V, a light-dependent DNA intercalating agent, shows promising therapeutic effects for psoriasis, and its light-activatable chemical probe derivative enabled the identification of 311 DNA-binding proteins which is significant for new biology discovery.

## INTRODUCTION

DNA damage occurs spontaneously and causes a plethora of human pathologies, including cancer and premature aging [1,2]. DNA adducts are one form of DNA damage, caused by the covalent modification with a chemical moiety. They can interfere with DNA replication, trigger cell-cycle arrest and ultimately induce programmed cell death [3]. Aberrant DNA structures caused by damaging agents can lead to cytotoxicity or induce cancer-causing mutations, but they are also induced by many chemotherapeutic agents. Direct DNA-damage-inducing agents, such as alkylating agents [4], platinum analogs [5] and antitumor antibiotics [6], are under constant investigation as a promising drug-development direction. However, despite their robust efficacy in cancer treatment, the treatment with such agents is often marred by drug-resistance problems at least in part due to DNA-damage repair [7].

The DNA-damage response (DDR) is a sophisticated signaling network, evolved to protect DNA integrity from endogenous or exogenous damage. Many different types of DNA damage are known, including modification of individual bases, intra-strand cross links, inter-strand cross links (ICLs), DNA–protein cross links (DPCs), single-strand breaks (SSBs) and double-strand breaks (DSBs) [8]. A variety of DNA-damage-repair pathways can be triggered in response to different DNA damages, including base excision repair, SSB repair, nucleotide excision repair (NER) [9], non-homologous end-joining, homologous recombination, etc. [10]. Targeting the DDR provides an essential strategy for cancer treatment by inducing synthetic lethality—a direction of effort for concomitant inactivation of two genes leading to cell death [11,12]. The combined usage of DDR inhibitors can augment the effect of DNA-damaging chemotherapy drugs and is a promising approach to reduce drug resistance [13]. Among current endeavors to target the DDR for cancer treatment, inhibitors against poly-ADP-ribose polymerase (PARP) [14], a DNA-damage sensor, have received Food and Drug Administration (FDA) approval in multiple ovarian cancer and breast cancer indications, while many other agents are currently undergoing clinical trials. Our current understanding of DDR is still incomplete [15,16]. Hence, new insights into the DDR with chemical approaches will facilitate drug discovery in this field.

**Figure 1.**
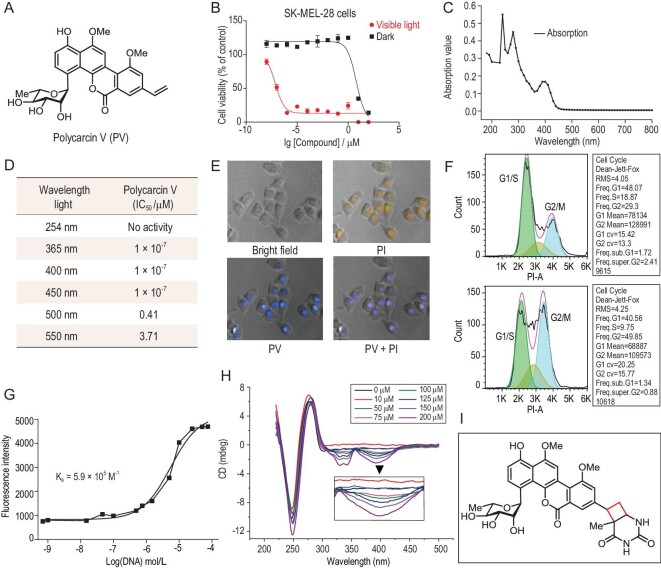
The light activates polycarcin V, covalently interacting with DNA. (A) Chemical structure of polycarcin V. (B) The light-dependent cytotoxicity of polycarcin V in SK-MEL-28 cells. (C) The light absorption spectrum of polycarcin V. (D) Wavelength screening for polycarcin V activation. (E) Cellular localization of polycarcin V. (F) Polycarcin V induces cell-cycle arrest at G2/M phase (top, DMSO; bottom, 1 nM polycarcin V for 24 h). (G) Fluorescence spectrum of polycarcin V-calf DNA interaction. (H) Circular dichroism spectrum of polycarcin V-DNA interaction. (I) [2+2] cycloaddition product of polycarcin V with thymine.

Some natural products are DNA-cross-linking agents, such as colibactin, associated with colorectal cancer formation in humans [17,18]. We have focused on the gilvocarcins, a group of secondary metabolites produced by Streptomyces that share an oxidatively rearranged polyketide-derived benzo[d]naphtha[1,2-b]pyran-6-one backbone decorated with a C-glycosidically linked sugar moiety [19–24]. These compounds exhibit remarkable light-activated antitumor activities [25] and their proposed mode of action is that they covalently cross-link to DNA upon ultraviolet (UV)-light irradiation [26,27]. Polycarcin V, one of the most potent gilvocarcins, is a C-aryl glycoside compound with an α-linked L-rhamnopyranose moiety [28,29] (Fig. 1A) and it also has the capacity of binding to DNA [30]. Our lab has developed a 10-step, scalable approach toward the synthesis of polycarcin V using the strategies of C–H functionalization and late-stage C-glycosylation [31]. Given its potential light-activated DNA-modifying activity, polycarcin V is a promising chemical that can be applied in light-based therapy as a novel DNA-damaging agent and developed into a DNA-cross-linking chemical probe to enable new biological discoveries.

Here, we show that polycarcin V is a light-activatable DNA-intercalating agent that causes DNA damage by covalently forming a [2+2] cycloadduct with thymine bases in DNA following irradiation with 365–450 nm of light in a sequence-independent manner. Based on the polycarcin V structure, we designed and synthesized a light-activatable cross-linking chemical probe to profile DNA-binding proteins via a chemoproteomic approach. We identified 311 DNA-binding proteins that were cross-linked to DNA by our probe. Among the isolated proteins were those involved in the NER pathway and we show that the bulky polycarcin V-DNA adducts are indeed repaired by NER, validating our approach. Additionally, we identified many novel DNA-binding proteins, candidates for further biological investigations and drug development. Finally, we evaluated polycarcin V for psoriasis treatment and proposed that the combinatorial therapies of polycarcin V with NER protein inhibitors may enhance the curative effect.

## RESULTS AND DISCUSSION

### Polycarcin V is a light-activatable DNA-damaging agent

To investigate whether polycarcin V is a light-activated natural product, we treated various cell lines, including SK-MEL-28, with polycarcin V under visible light or in the dark. Subsequently, we assessed the cell viability using a CellTiter-Glo assay. The cytotoxicity increased by several orders of magnitude upon exposure to light (Fig. 1B and Table S1), suggesting that light is necessary for the potent bioactivity of polycarcin V. With light irradiation, a low dose of polycarcin V induced caspase-dependent cell death (Fig. S1). Furthermore, the UV/VIS spectrum indicates that polycarcin V absorbs light at a wavelength of 250–450 nm (Fig. 1C) and light at a wavelength of 365–450 nm is effective for polycarcin V activation, as suggested by the assay correlating the wavelength with toxicity (Fig. 1D).

To confirm the mode of action of polycarcin V, we first determined its cellular localization. We found that it accumulated in the nucleus (Fig. 1E), consistently with an interaction of polycarcin V with DNA. Fluorescence activating cell sorter (FACS) analysis revealed that upon polycarcin V treatment, the cell cycle was arrested at the G2/M phase (Fig. 1F). Additionally, the fluorescence spectrum assay demonstrated strong interaction between polycarcin V and calf thymus DNA, with a Ka of ∼5.9 × 10^5^ M^–1^ (Fig. 1G and Fig. S2). We monitored the circular dichroism spectrum [32] of polycarcin V incubated with DNA to determine its DNA-binding mode. A characteristic negative peak at 375 nm suggested that polycarcin V is a DNA-intercalating agent (Fig. 1H). Finally, we isolated the thymine–polycarcin V adduct and characterized it using mass spectrometry (MS) and nuclear magnetic resonance (NMR) (Fig. S3). We discovered that a covalent [2+2] photoadduct was generated between the vinyl group of polycarcin V and a thymine residue of DNA (Fig. 1I). Altogether, our data indicate that polycarcin V causes DNA damage by forming a covalent [2+2] cycloadduct with a thymine residue following exposure to 365–450 nm of light.

### Polycarcin V modifies DNA without sequence specificity

**Figure 2.**
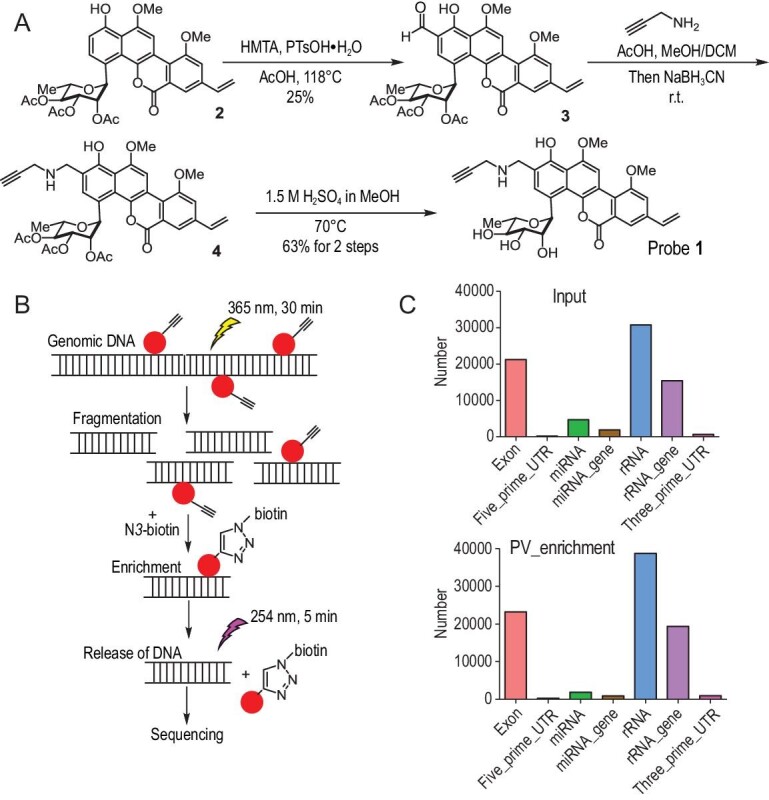
High-throughput sequencing of polycarcin V modification on genomic DNA. (A) Synthesis of probe **1** for high-throughput sequencing. (B) Workflow of sample preparation for high-throughput sequencing. (C) The distribution of reads from input and polycarcin V enrichment samples in different regions of the reference genome.

To develop polycarcin V as a DNA-cross-linking chemical probe, it is necessary to map the global interactions of this compound with chromatin in a genome-wide fashion. First, we synthesized a primary probe **1**, an alkynylated derivative of polycarcin V (Fig. 2A), to perform the Chem-seq. Using acyl protected polycarcin V (Compound **2** in Fig. 2A) as the starting material, a formyl group was first installed at the C2 position using a Duff reaction to afford intermediate **3** with 25% yield, followed by reductive amination to yield alkyne **4**. Finally, using 1.5 M of H_2_SO_4_ in MeOH cleanly, acid-induced deacetylation afforded the desired probe **1** (63% for two steps). The potency of probe **1** to cross-link DNA was confirmed by an *in vitro* binding assay (Fig. S4). To profile the DNAs bound by probe **1** using high-throughput sequencing (Fig. 2B), we treated HeLa cells with 20 μM of probe **1** for 1 hour and then irradiated the cells under 450 nm of light to cross-link probe **1** to genomic DNA. Genomic DNA was extracted and fragmented via sonication. Probe-bound DNAs were enriched by attaching N_3_-biotin to the probe using click chemistry and subsequent pull-down with streptavidin beads. Finally, the DNA samples were irradiated at 254 nm to reverse probe–DNA cross links (Fig. S5), followed by library preparation for massively parallel DNA sequencing. We identified 14 471 peaks that were evenly distributed across the chromosomes. Additionally, no significant motifs were identified by statistical analysis of the peak sequences. Compared to the input DNAs sample, the DNAs pulled down by polycarcin V showed no considerable enrichment genomic regions (Fig. 2C). In conclusion, genome-wide mapping reveals that polycarcin V cross-links to DNA independently of its sequence and causes widespread damage to genomic DNA. Therefore, polycarcin V could serve as a good prototype for developing a DNA-cross-linking chemical probe.

### A light-activated chemical probe was designed and synthesized

**Figure 3.**
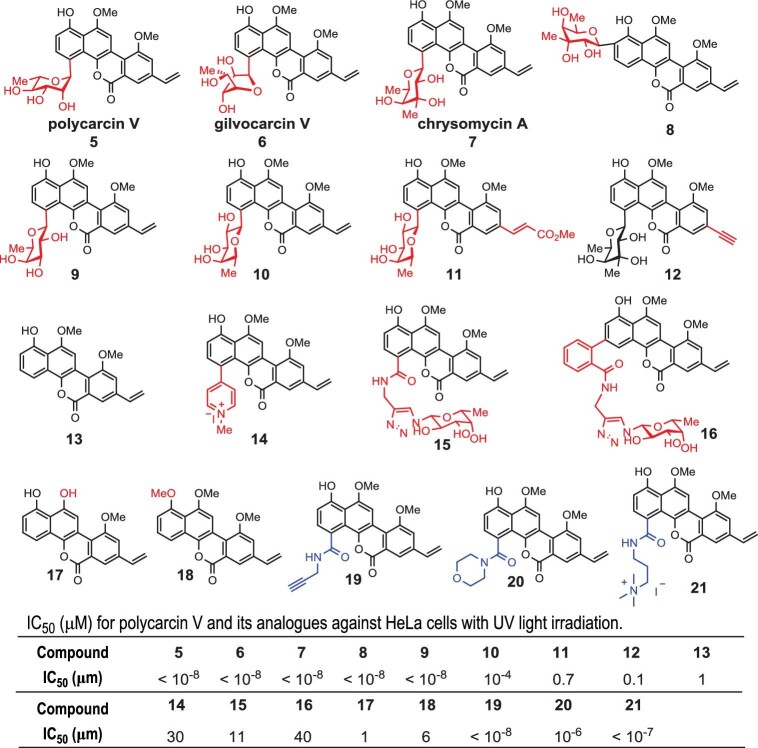
Photoactivated antitumor activity of polycarcin V analogs. HeLa cells were treated with polycarcin V and analogs followed by 365 nm of irradiation for 20 min. After 48 h of continuous cultivation, the IC_50_ values were determined by CellTiter-Glo assay. (Note: some analogs have been published in our previous article [31].)

To simplify the structure of the chemical probe, we broadly evaluated the anticancer activity of the polycarcin V derivative library (Fig. 3). Polycarcin V, gilvocarcin V and chrysomycin A all showed potent photoactivated antitumor activity, C2 glycosylated derivative **8** and C4 glycosylated derivative **10** also showed total activity. However, derivatives **11**–**18** lost activity. Interestingly, we also found that simple amide groups could replace the complex glycosidic moiety while preserving the activity (Fig. 3 and Fig. S6). Compound **19** with a propargyl amide group in the C4 position of the aglycon showed comparable activity to polycarcin V. Compounds **20** and **21** also showed similar activity. Hence, we designed a bifunctional moiety carrying both a diazirine group (for photo-cross-linking with proteins) and a terminal alkyne (for conjugation with azide-biotin, allowing enrichment streptavidin beads) to be installed at the aglycan part of polycarcin V through amide formation. We expected that such derivatization could facilitate the covalent cross-linking of DNA with the vinyl group upon blue-light activation and subsequent conjugation with proteins nearby with the diazirine group upon UV activation. Two-step activation of such a chemical probe thus could cross-link protein residues with DNA, setting the stage for identifying and analysing proteins that bind close to the DNA lesions generated by polycarcin V and proteins that dynamically and weakly interact with DNA.

**Figure 4.**
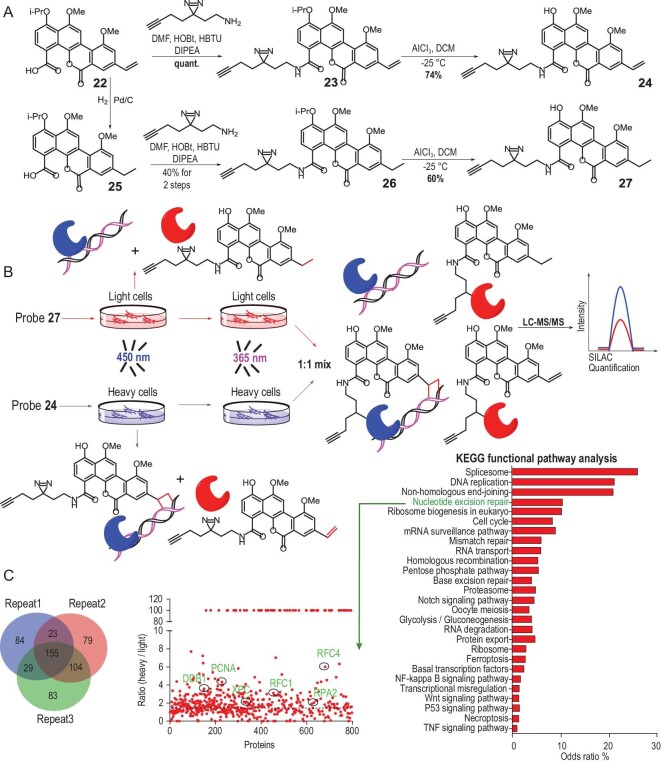
MS-based profiling of DNA-interacting proteins in living cells by ABPP-SILAC. (A) Synthesis of positive (**24**) and negative (**27**) cross-linking chemical probes. (B) Workflow of ABPP-SILAC. (C) Statistics and KEGG functional pathway analysis of DNA-interacting proteins.

Leveraging one of the critical intermediates from our synthesis of polycarcin V derivatives, we synthesized the designed probe **24** in only two steps (amide formation, deprotection) (Fig. 4A). Negative control for the probe (**27**) with the DNA-reductive alkene removed was also synthesized to rule out the background signals. Probe **24** instead of control compound **27** maintained bioactivity as demonstrated by cytotoxicity assay (Fig. S7).

### MS-based activity-based protein profiling of DNA-binding proteins in living cells

We next utilized the chemical probe via a chemoproteomic approach [33,34] to identify DNA-binding proteins in living cells. Activity-based protein profiling (ABPP) reveals the target proteins in the complex biological system using the active site-directed probes [35]. To perform the ABPP-SILAC (stable isotope labeling with amino acids in cell culture) experiment, MCF-7 cells were cultured in ‘light’ and ‘heavy’ SILAC media for several passages until the metabolic incorporation of heavy amino acids was complete. Subsequently, the light and heavy cells were treated with a 20-μM positive (**24**) or control (**27**) probe and irradiated with 450 nm of light to cross-link to DNA and successively 365 nm of light to cross-link any adjacent proteins. Cells were lysed and combined in a 1 : 1 ratio, followed by conjugating the alkynyl groups of **24** and **27** to an azide-biotin tag by a CuAAC-catalysed click reaction. After enrichment with streptavidin and digestion by trypsin, the peptides of the proteomic were analysed by mass-spectrometric (LC-MS/MS) and the SILAC ratio was quantified for each protein (Fig. 4B). According to the MS data of three independent experiments, we found 311 DNA-binding protein candidates enriched by more than 1.5-fold for the positive probe **24** in at least two experiments (Fig. 4C), demonstrating the specificity of our assay. Among them were proteins involved in NER (XPC, DDB1, DDB2) and those involved in replication and DNA-repair synthesis (PCNA, RFC1, RFC4) (Fig. 4C). NER is the main pathway for repairing bulky, helix-destabilizing lesions. We hypothesized that the [2+2] DNA cycloadduct modified with polycarcin V, considered a bulky DNA adduct, could be repaired by NER. Aside from some known DNA-binding proteins, including DNA-replication proteins, DNA helicases, topoisomerases, etc., a large number of proteins that have not been previously associated with DNA binding or DNA-damage repair were also cross-linked by our probes, such as serine/threonine-protein kinases, E3 ubiquitin-protein ligases, serine/threonine-protein phosphatases, etc., which warrant further investigation and could lead to the discovery of novel players in DNA-damage repair and unknown DNA–protein interaction.

### The [2+2] photoadduct is repaired by NER

**Figure 5.**
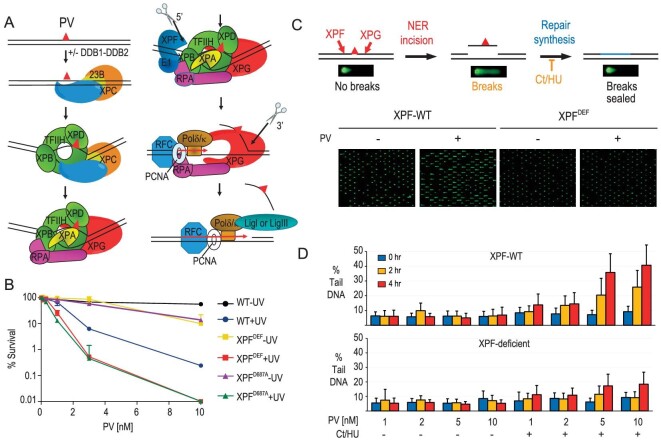
Nucleotide excision repair (NER) repairs polycarcin V-DNA adducts. (A) Proposed model of NER system-mediated repair of the polycarcin V-DNA [2+2] cycloadduct. (B) Clonogenic survival assays: XP2YO Cells (XPF-proficient expressing no, XPF-WT or catalytically inactive XPF-D687A) were treated with polycarcin V (PV) and UV (365 nm) and the surviving fraction was determined. The data were plotted as the percentage of colonies formed on plates of treated cells versus the untreated control. (C) Assessment of NER incision activity using alkaline CometChip assays. (D) Quantifying percentage of DNA in comet tail, representing DNA gaps, in cells exposed to the indicated doses of PV ± CT/HU. DNA tail lengths were measured at 0, 2 and 4 h after PV/UV treatment.

To determine whether the polycarcin V-DNA [2+2] cycloadduct is indeed repaired by NER (Fig. 5A) and validate the feasibility of this bifunctional chemical probe, we performed the clonogenic survival assays. We treated XPF-deficient XP2YO cells complemented with no protein, XPF-WT (wild type) or catalytically inactive XPF-D687A protein with increasing concentration of polycarcin V, irradiated with 365 nm of light. We measured the surviving fraction of cells using clonogenic assays. Compared with WT cells, the NER-deficient cells (XPF-deleted and catalytically inactive XPF-D687A) displayed an apparent hypersensitivity to polycarcin V, suggesting that polycarcin V-DNA adducts were repaired by NER (Fig. 5B)—an observation confirmed in cells deficient in XPA, another essential NER protein (Fig. S8). We utilized a NER-specific alkaline CometChip assay to monitor NER incision activity directly. In NER, a dual incision around a lesion leads to the excision of an oligonucleotide containing the damage of 25–30 nucleotides in length and the formation of a gap. The gap is filled in by repair synthesis. This step can be inhibited by adding cytarabine and hydroxyurea, yielding persistent gaps that can be detected as DNA with single-stranded breaks in alkaline comet assays [36]. Following treatment with polycarcin V ± 365 nm of light, we observed a dose-dependent increase in the gap formation using COMET CHIP assays in XPF-WT and XPA-WT expressing cells, but not the corresponding XPF- and XPA-mutant cells (Fig. 5C and D, and Fig. S8B). Quantifying the % DNA in a comet tail representing DNA gaps in cells revealed that gap formation depended on polycarcin V, CT/HU and NER proficiency, demonstrating that NER incises the [2+2] cycloadduct. These observations validated that the [2+2] DNA cycloadduct was indeed repaired by the NER pathway and further validated our chemical probe's reliability for DNA-binding-protein profiling.

### Potential therapeutic application in psoriasis

In addition to serving as the basis for chemical cross-linking probe development, polycarcin V and its derivatives also have potential applications in photodynamic therapy, especially the treatment of skin disorders, where light penetration can be more easily achieved. Psoriasis is a chronic inflammatory skin disease caused by immune-system dysfunction and decreased quality of life. Characteristic symptoms of psoriasis include erythema, scales and increased skin thickness [37,38]. PUVA (psoralen and ultraviolet A) therapy is a treatment for psoriasis in the clinic, which typically uses the DNA-intercalating agent 8-methoxypsoralen (8-MOP) to sensitize the skin plus exposure to ultraviolet A (UVA) radiation to induce apoptosis of skin cells [39,40]. However, UV light is known to increase the risk of skin cancers. The adverse effects of UV could be avoided if a higher wavelength of light, such as blue light, could be used to replace UV for psoriasis treatment.

**Figure 6.**
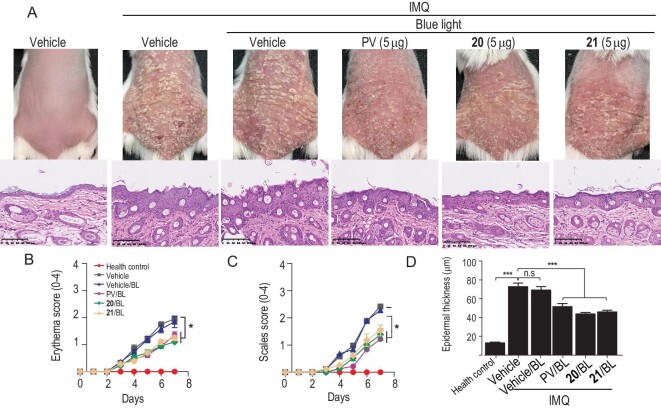
Anti-psoriasis efficacy of polycarcin V and its derivatives 20 and 21. Following the same procedure to test the anti-psoriasis efficacy of polycarcin V derivatives (*n* = 6, female, 8 weeks old). (A) The representative phenotype of back skin in each group after designed treatment (up) and the hematoxylin-eosin (H&E) staining sections of skin tissues after tissues collection (down). Scale, 100 μm. (B) and (C) The scales and erythema scores were monitored daily, following clinical psoriasis area and severity index: erythema and scales are calculated respectively on a scale from 0 to 4: 0, none; 1, slight; 2, moderate; 3, marked and 4, highly marked. (D) The epidermal thickness quantitative data are based on pathological sections. Data are shown as the mean ± s.e.m. of respective *n* biologically independent samples. One-way analysis of variance (ANOVA) determined *P*-values with Tukey's multiple comparison post hoc test. n.s., no significance. *P < 0.05; **P < 0.01; ***P < 0.001 versus vehicle group or indicated in the figures. PV, polycarcin V; BL, blue light.

In terms of both bioactivity and binding affinity with DNA, the activity of polycarcin V is several orders of magnitude higher than 8-MOP (Fig. S9A and B). Notably, the wavelength of light needed for polycarcin V activation (blue light) is much safer than that for 8-MOP (UV light) (Fig. S9D). To evaluate the potential application of polycarcin V and its derivatives in psoriasis treatment, we examined their anti-psoriasis effects following irradiation with 365 nm of blue light (440–470 nm) in animal models. Topical application of imiquimod (IMQ) cream on the skin of mice is a widely used model for mimicking psoriasis symptoms. After daily application of IMQ cream (62.5 mg/mouse/day) on mice backs for 7 days, the skin demonstrated psoriasis-like lesions with increased epidermal thickness, thick scales and erythema. Under 365 nm-mediated photodynamic therapy, external application of a tincture of polycarcin V significantly alleviated the psoriasis features of skin lesions, based on the scores for scales, erythema and thickness. It also showed comparable therapeutic efficacy under the same dose when compared to positive control, 8-MOP (Fig. S10A–E). Under blue-light-mediated photodynamic therapy, tincture of polycarcin V external application also significantly alleviated psoriasis skin lesions, showing better therapeutic efficacy than 8-MOP at the same and lower doses (Fig. S11A–E). Besides, the anti-psoriasis efficacy of polycarcin V under blue-light-mediated photodynamic therapy was dose-dependent. Polycarcin V derivatives **20** and **21** showed anti-psoriasis efficacy after blue-light activation in IMQ-induced psoriasis mice models (Fig. 6A–D). Brief blue-light exposure of <1 min is enough to activate and induce such an anti-psoriasis effect (Fig. S12A–D). We also tested the pharmacokinetics parameters of polycarcin V and **20** in mice (Table S2). No adverse events were found (daily activities, body weight, organ/body weight ratio) of **20** in mice after an acute toxicity test (maximum tolerated dose, 2000 mg/kg, P.O. (peros)) (Fig. S13A and B). These results suggest that photodynamic treatment combining blue light and polycarcin V or its drug-like derivatives effectively alleviates IMQ-mediated psoriasis-like skin disorders without acute side effects in mice, providing a promising new therapeutic strategy for treating psoriasis effectively and safely.

## CONCLUSION

We illustrated the mode of action of a DNA-damaging agent, polycarcin V. We developed a polycarcin V-based light-activatable chemical cross-linking probe to investigate the DDR and unknown dynamic and weak DNA-binding proteins. Three hundred and eleven DNA-binding protein candidates, including proteins involved in the NER pathway, such as DDB1, PCNA, XPC, RFC1, RFC4 and XPA, were identified utilizing this probe. Pre-clinical studies with the psoriasis mouse model suggested that polycarcin V and its drug-like derivatives provide promising photosensitizers for skin-disease treatment. The proposed treatment combined with an NER inhibitor would improve the therapeutic efficacy. The characterization of polycarcin V and the novel polycarcin V-based cross-linking chemical probe presented here lay a foundation for future development of light-controlled drugs and provide a new tool for further research in the field of DNA–protein interactions and DDR biology. The novel DNA-binding proteins identified in this study are potentially novel components of DNA-damage-repair pathways and may serve as candidates for new drug development.

## METHODS AND MATERIALS

The experimental details are given in the online Supplementary data.

## Supplementary Material

nwac046_Supplemental_File
