# Supplementary material for: Polycarcin V induces DNA-damage response and enables the profiling of DNA-binding proteins
Source: Natl Sci Rev. 2022 Mar 11;9(11):nwac046. doi: 10.1093/nsr/nwac046 (PMC9798893; doi:10.1093/nsr/nwac046)
Supplement: nwac046_Supplemental_File [file nwac046_supplemental_file.pdf]

Polycarcin V induces DNA damage response and enables the profiling of DNA-binding proteins

Zongwei Yue[1†], Fan Wu[1†], Fusheng Guo[1,2†], Jiyeong Park[3], Jin Wang[1], Liyun Zhang[1], Daohong Liao[5], Wenyang Li[1], Orlando D. Schärer[3,4], Xiaoguang Lei[1,2,6*]

[1] Beijing National Laboratory for Molecular Sciences, Key Laboratory of Bioorganic Chemistry and Molecular Engineering of Ministry of Education, Department of Chemical Biology, College of Chemistry and Molecular Engineering, Synthetic and Functional Biomolecules Center, Peking University, Beijing, 100871, China

[2] Peking-Tsinghua Center for Life Science, Academy for Advanced Interdisciplinary Studies, Peking University, Beijing 100871, People's Republic of China

[3] Center for Genomic Integrity, Institute for Basic Science, Ulsan, 44919, Republic of Korea

[4] Department of Biological Sciences, School of Life Sciences, Ulsan National Institute of Science and Technology, Ulsan, 44919, Republic of Korea

[5] Jiangsu JITRI Molecular Engineering Inst. Co., Ltd., Jiangsu 215500, People's Republic of China

[6] Institute for Cancer Research, Shenzhen Bay Laboratory, Shenzhen, 518107, China

[†] Contributed equally to this work.
* Corresponding authors. Email: xglei@pku.edu.cn (X.L.)

Supplementary Information

Table of Contents

**I) General Information**	S2
**II) Supporting Tables and Figures**	S3–S10
**III) Biochemical Experiments**	S11-S16
**IV) Chemical Synthesis**	S17–S25
**V) NMR Spectra**	S26–S35

I) General Information

^{1}H NMR spectra were recorded on Brucker ARX 400 MHz or DRX 500 MHz spectrometer at ambient temperature with $CDCl_3$, Methanol-d4 and MeCN-d3 as the solvent unless otherwise stated. ^{13}C NMR spectra were recorded on Brucker ARX 100 MHz or DRX 125 MHz spectrometer (with complete proton decoupling) at ambient temperature. Chemical shifts are reported in parts per million relative to chloroform, Methanol-d4 and MeCN-d3. Data for ^{1}H NMR are reported as follows: chemical shift, integration, multiplicity (s = singlet, d = doublet, t = triplet, q = quartet, m = multiplet) and coupling constants. Infrared spectra were recorded on a Thermo Fisher FT-IR200 spectrophotometer. High-resolution mass spectra were obtained at Peking University Mass Spectrometry Laboratory using a Bruker APEX Flash chromatography. Analytical thin layer chromatography was performed using 0.25 mm silica gel 60-F plates. Flash chromatography was performed using 200–300 mesh silica gel. Yields refer to chromatographically and spectroscopically pure materials unless otherwise stated. Dichloromethane, dichloroethane, acetonitrile and dimethyl formamide were distilled from calcium hydride; tetrahydrofuran was distilled from sodium/benzophenone ketyl prior to use. Reagents were purchased at the highest commercial quality and used without further purification unless otherwise stated. All reactions were carried out in oven-dried glassware under an argon atmosphere with dry solvents unless otherwise noted.

II) Supporting Tables and Figures

Cell line	Visible Light (IC_{50})	Dark (IC_{50})
**HeLa**	< 0.01 pM	6 μM
**HCT116**	~0.01 pM	50 μM
**MCF-7**	0.5 pM	8 μM
**HEPG2**	< 0.01 pM	5 μM
**KB**	0.3 pM	4 μM
**SK-MEL-28**	0.2 pM	6 μM
**A549**	6 pM	65 μM
**MDA-MB-231**	< 0.01 pM	9 μM
**K562**	< 0.01 pM	3 μM
**Hut-78**	760 pM	> 100 μM
**HaCat**	< 0.01 pM	6 μM

Table. S1. The light-dependent activity of polycarcin V against different tumor cell lines. Various tumor cell lines were treated with different concentrations of polycarcin V under visible light or dark. Cell visibility and IC_{50} value were determined by cell titer glo kit.

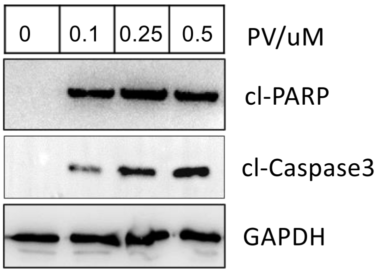

Figure S1. Polycarcin V activates caspase-dependent cell death pathway. With polycarcin V treatment for 24 h, the expression of cl-PARP and cl-Caspase 3 were both activated.

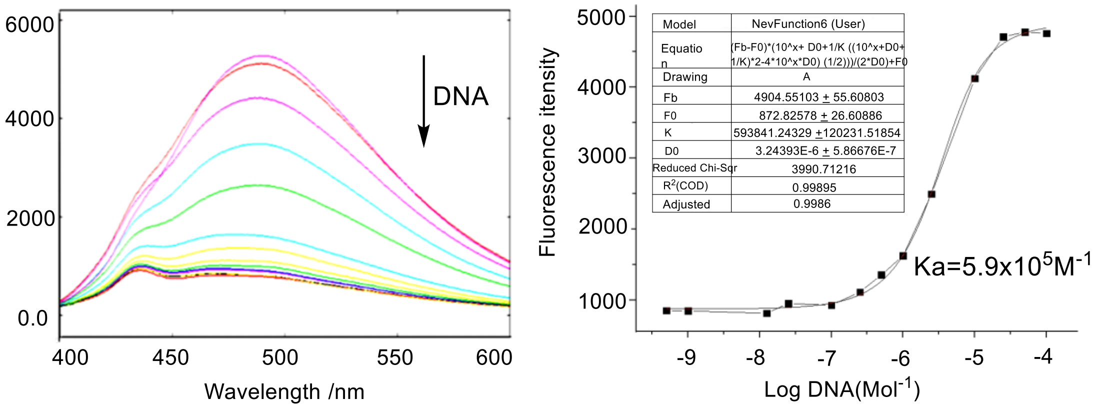

Figure S2. Fluorescence spectra for polycarcin V interacts with calf-DNA in dark. Fluorescence spectra and Least Squares Fitting analysis of polycarcin V in the presence of varying concentrations of CT-DNA, [Polycarcin V] =3.9 x 10^{-7} mol L^{-1}, [CT-DNA] = 0-5.00 x 10^{-7} mol L^{-1}.

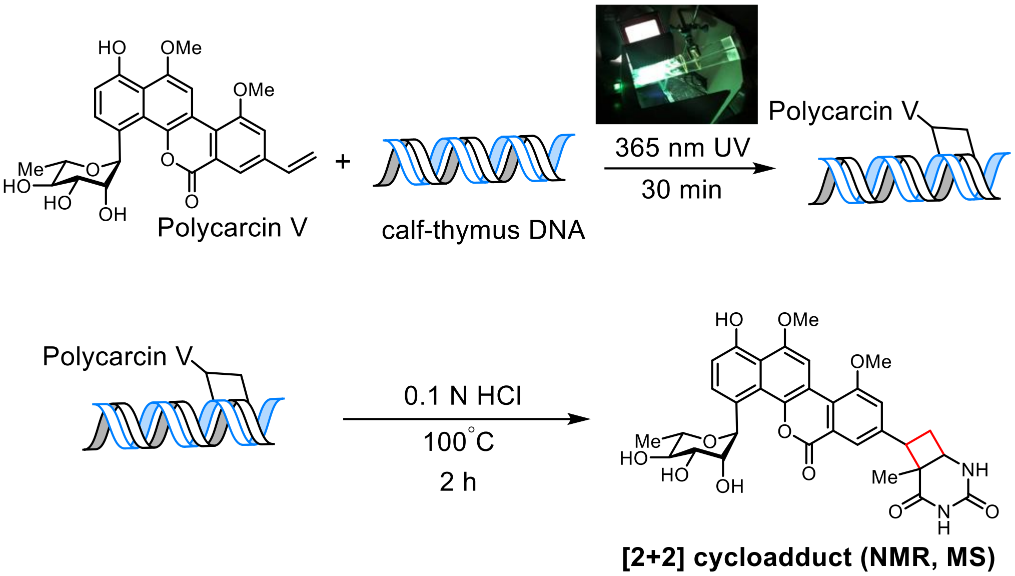

Figure S3. Polycarcin V forms [2+2] cycloadduct with DNA thymine residue. Polycarcin V were incubated with calf-DNA with 365 nm UV irradiation for 30 min. The [2+2] cycloadduct was isolated by acid hydrolysis in high temperature and characterized by MS and NMR.

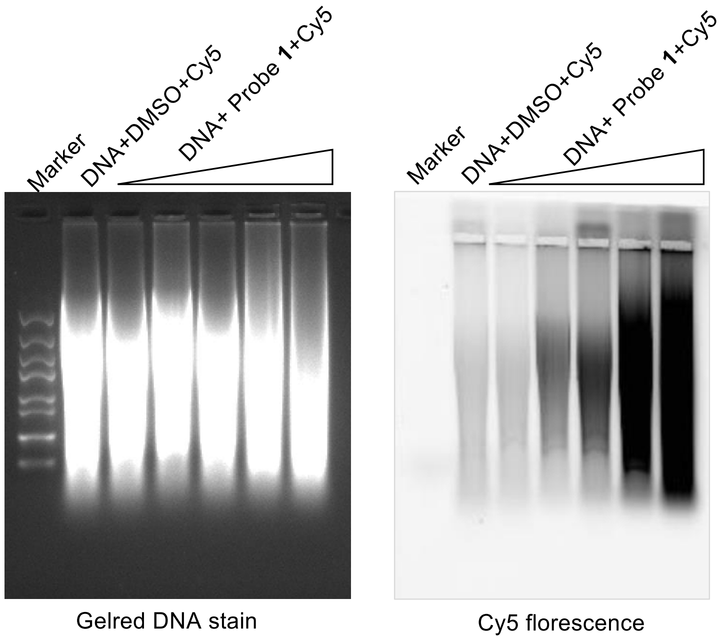

Figure S4. Probe 1 interacts with calf-DNA in vitro. Increasing concentrations of probe **1** was incubated with 10 μg calf-DNA under 365 nm light for 30 min. The

purified probe 1-DNA was conjugated with fluorochrome Cy5 by click reaction, and analyzed by DNA gel electrophoresis.

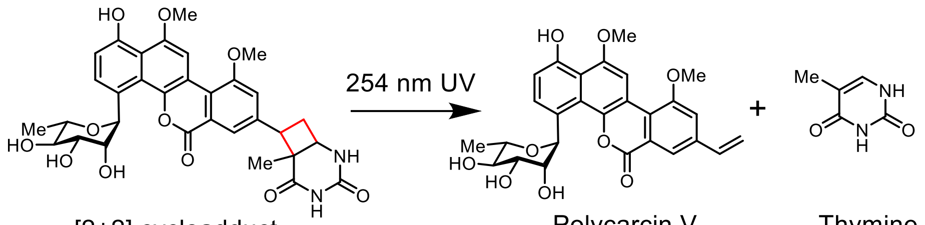

[2+2] cycloadduct Polycarcin V Thymine

Figure S5. The reverse reaction of [2+2] cycloadduct under 254 nm UV light. Upon 254 nm UV irradiation for 10 min, the [2+2] adduct was hydrolyzed as polycarcin V and thymine.

a.

compound	IC_{50} (μM)		
	HeLa	HEPG2	HaCat
**Polycarcin V**	1×10^{-8}	$< 10^{-8}$	$< 1\times10^{-8}$
**20**	1×10^{-6}	4×10^{-8}	10^{-8}

b.

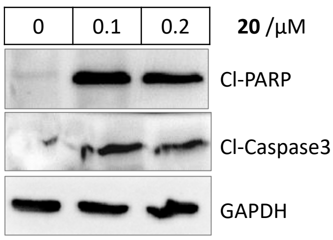

Figure S6. 20 induces caspase-dependent apoptosis. a. HeLa cells, HEPG2 and HaCat cells were treated with polycarcin V and compound **20** followed by 450 nm irradiation for 20 min. After 48 h of continuous cultivation, the IC_{50} values were determined by celltiter glo kit. **b.** Upon I-18 treatment and 450 nm light irradiation, the biomarker protein cl-PARP and cl-Caspase3 were up-regulated.

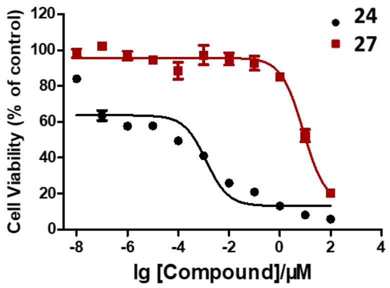

probe	IC_{50}(μM)
**24**	$1X10^{-4}$
**27**	10

Figure S7. Evaluation of bioactivity of positive probe 24 and negative probe 27. HeLa cells were treated with probe **24** and **27**, followed by 450 nm light irradiating for 20 min. IC_{50} value was determined by celltier glo kit.

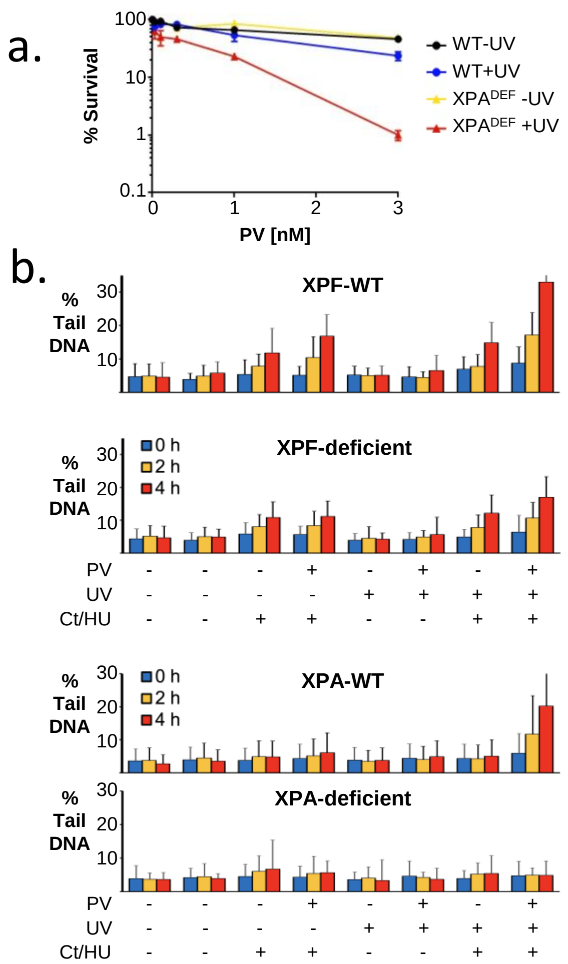

Figure S8. Polycarcrin V DNA adducts are repaired by Nucleotide Excision Repair. **a**. Clonogenic survival assays: XP2OS Cells (XPA-deficient expressing no or XPA-WT) were treated with polycarcin V (PV) and UV (365 nm) and the surviving fraction determined. The data were plotted as the percentage of colonies formed on plates of treated cells versus the untreated control. **b.** Assessment of NER incision activity using alkaline CometChip assays in XP2YO cells (XPF-proficient expressing no or XPF-WT) and XP2OS Cells (XPA-deficient expressing no or XPA-WT). Quantification of % DNA in comet tail representing DNA gaps in cells exposed or not to 2 nM PV ± CT/HU. DNA tails lengths were measured 0 h, 2 h and 4 h after PV/UV treatment.

a.

compound	IC_{50} (μM)	
	HeLa	Hut-78
Polycarcin V	< 10^{-8}	7.6×10^{-4}
8-MOP	0.7	> 100

b.

compound	Binding constant Ka (M^{-1})
Polycarcin V	5.9×10^{5}
8-MOP	770$^{(2)}$

c.

Wavelength light	Polycarcin V (IC_{50}/ μM)	8-MOP (IC_{50}/ μM)
365 nm	1x 10^{-7}	0.8
400 nm	1x 10^{-7}	~100
450 nm	1x 10^{-7}	>100
500 nm	0.41	>100
550 nm	3.71	>100

Figure S9. Comparison of Polycarcin V and 8-MOP in the aspects of antitumor activity, DNA binding capacity, the required wavelength. a. HeLa and Hut-78 cells were treated with polycarcin V and 8-MOP followed by 365 nm light irradiation for 20 min. After 48 h of continuous cultivation, the IC_{50} values were determined by celltiter glo kit. **b.** The binding affinity with DNA were determined by fluorescence spectra assay. **c.** SK-MEL-28 cells were treated with polycarcin V and 8-MOP followed by different wavelength light irradiation for 20 min. After 48 h of continuous cultivation, the IC_{50} values were determined by celltiter glo kit.

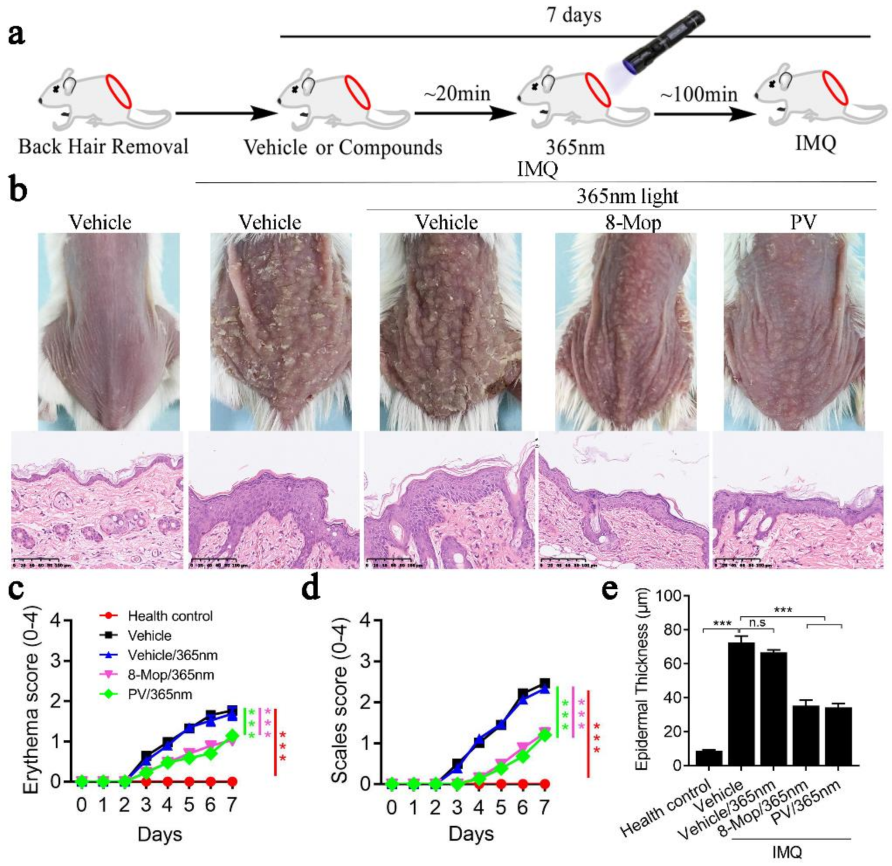

Figure S10. Anti-psoriasis efficacy of polycarcin V with UV-mediated photodynamic therapy. Efficacy of polycarcin V with 365nm-mediated photodynamic therapy on IMQ-induced psoriasis treatment. **a.** Experimental design of IMQ-induced psoriasis mice model and treatment arrangement (n = 6, female, 8 weeks old). **b.** Representative phenotype of back skin in each group after designed treatment (up) and the H&E staining sections of skin tissues after tissues collection (down). Scale, 100 μm.

c-d. The scales and erythema scores were monitored daily, following clinical psoriasis area and severity index (PASI): erythema and scales are calculated respectively on a scale from 0–4: 0, none; 1, slight; 2, moderate; 3, marked and 4, highly marked. **e.** The epidermal thickness quantitative data based on pathological sections. Data are shown as the mean ± s.e.m. of respective n biologically independent samples. P values were determined by one-way ANOVA with Tukey's multiple comparison post hoc test. n.s, no significance, *P < 0.05, **P < 0.01 and ***P < 0.001 versus vehicle group or indicated in the figures.

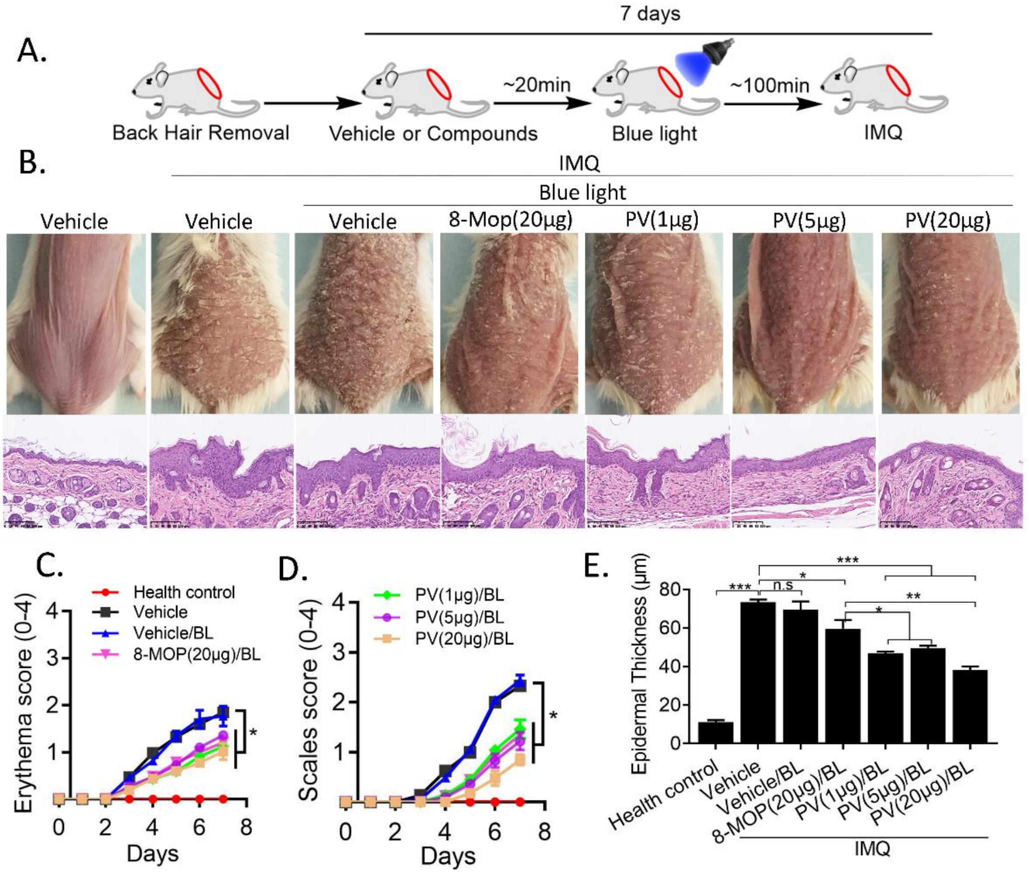

Figure S11. Anti-psoriasis efficacy of polycarcin V with blue light-mediated photodynamic therapy. Efficacy of polycarcin V with blue light-mediated photodynamic therapy on IMQ-induced psoriasis treatment. (A) Experimental design of IMQ-induced psoriasis mice model and treatment arrangement (n = 6, female, 8 weeks old). (B) Representative phenotype of back skin in each group after designed treatment (up) and the H&E staining sections of skin tissues after tissues collection (down). Scale, 100 μm. (C, D) The scales and erythema scores were monitored daily, following clinical psoriasis area and severity index (PASI): erythema and scales are calculated respectively on a scale from 0–4: 0, none; 1, slight; 2, moderate; 3, marked and 4, highly marked. (E) The epidermal thickness quantitative data based on pathological sections. Data are shown as the mean ± s.e.m. of respective n biologically independent samples. P values were determined by one-way ANOVA with Tukey's multiple comparison post hoc test. n.s, no significance, *P < 0.05, **P < 0.01 and ***P < 0.001 versus vehicle group or indicated in the figures.

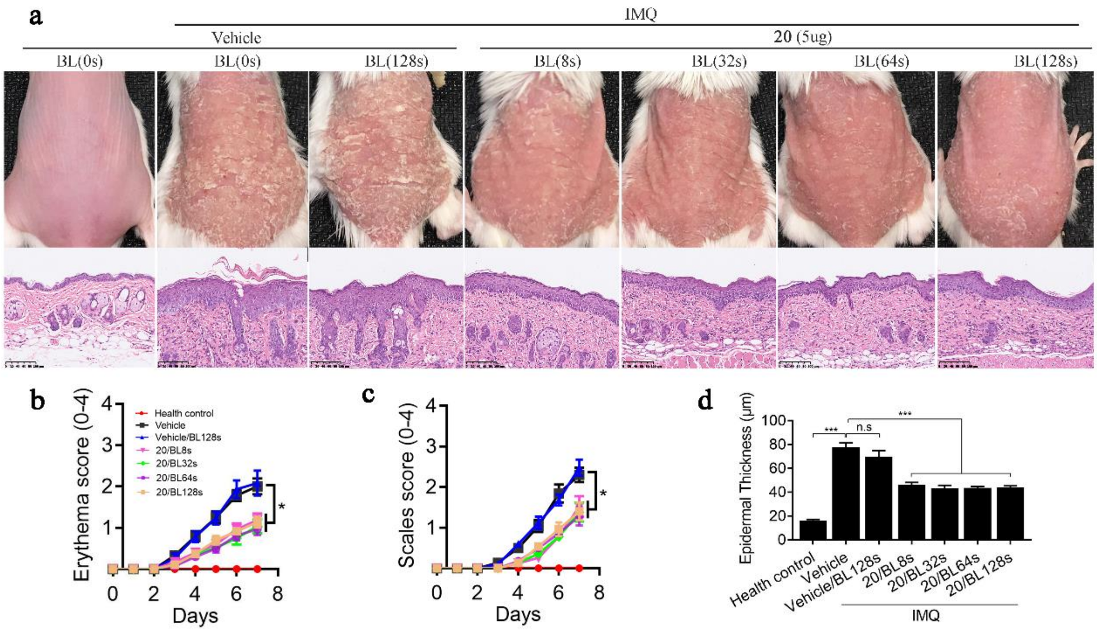

Figure S12. Anti-psoriasis efficacy of 20 with blue light gradient exposure. Following the above-mentioned same procedure to test the anti-psoriasis efficacy of **20** under different blue light exposure time (n = 6, female, 8 weeks old). **a.** Representative phenotype of back skin in each group after designed treatment (up) and the H&E staining sections of skin tissues after tissues collection (down). Scale, 100 μm. **b-c.** The scales and erythema scores were monitored daily, following clinical psoriasis area and severity index (PASI): erythema and scales are calculated respectively on a scale from 0–4: 0, none; 1, slight; 2, moderate; 3, marked and 4, highly marked. **d.** The epidermal thickness quantitative data based on pathological sections. Data are shown as the mean ± s.e.m. of respective n biologically independent samples. P values were determined by one-way ANOVA with Tukey's multiple comparison post hoc test. n.s, no significance, *P < 0.05, **P < 0.01 and ***P < 0.001 versus vehicle group or indicated in the figures. Abbreviation, BL, blue light.

	$t_{1/2}$	T_{max}	C_{max}	AUC $_{0\text{-}t}$	AUC $_{0\text{-}inf}$	Vz	Cl	MRT $_{0\text{-}inf}$
	h	h	ng/mL	ng/mL*h	ng/mL*h	(mg)/(ng/mL)	(mg)/(ng/mL)/h	h
PV	1.27	-	125	108.4	115.2	0.0032	0.0018	1.42
SD	0.03	-	4.25	9.2	11.9	0.0004	0.0002	0.31
**20**	2.49	-	645.5	425.5	480.6	0.0047	0.0013	2.20
SD	0.25	-	256.2	96.9	100.0	0.0012	0.0002	0.39

Table. S2. Pharmacokinetics parameters. The pharmacokinetics parameters of compound polycarcin V and **20** were tested by a single intravenous (i.v.) in 8 weeks old male BABL/C mice (n = 3 group) at a dose of 1 mg/kg and 3 mg/kg. Blood samples were collected at 0.083, 0.25, 1, 2, 4, 6, 8, and 24 h post-administration through orbital venous plexus by capillary, EDTA-K2 anticoagulant. The blood samples were quantified by LC-MS/MS, and data analysis was conducted using PKsolver 2.0. Tmax, time of maximum plasma concentration; Cmax, maximum plasma concentration; AUC, area under the curve (measure of exposure); t1/2, half-life; Cl, plasma clearance; Vz, volume of distribution; MRT, mean residence time.

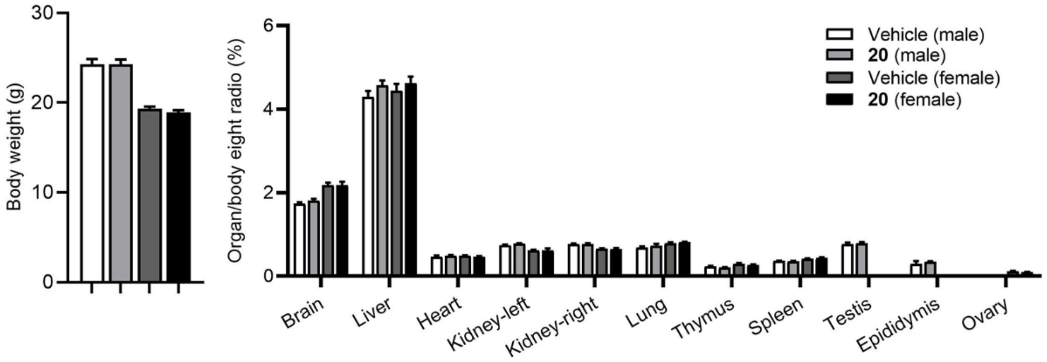

Figure S13. Acute toxicity test of 20 in mice. BABL/C mice (8 weeks, half male and half female, n = 10) were treated with single compound **20** (2000mpk) by gavage (p.o.), all animals were observed for 14 consecutive days to preliminarily test whether compound **20** own acute toxicity in mice. **a.** Body weight of control and single **20** treatment groups. **b.** The main organ/body weight radio in different groups.

III) Biochemical Experiments

Cytotoxicity assay. 1×10^4 tumor cells were seeded on 96-well plates and cultured in 37 ℃, 5% CO_2 for 12 h, followed by treatment with polycarcin V at indicated concentrations from 100 to 1×10^{-8} μM and 0 μM. Half an hour later, the. Cells were irradiated by viable light for 30 min or directly cultured in dark for 48 h. Cell viability was determined by celltiter-glo kit according to the manufacturer's instruction.

Western blot. HeLa cells in 10 cm dishes were treated with polycarcin V at 0, 0.1, 0.25, and 0.5 μM, followed by irradiated by 365 nm light for 30 min, and continue to culture for 24 h. The cells were lysed by 100 μL RIPA buffer with protease inhibitors, and detected by 12% SDS-PAGE with indicated antibodies immunoblotting. Cleaved PARP (Asp214) (D64E10) Rabbit mAb (#5625, Cell signaling), Cleaved Caspase-3(Asp175)(5A1E) Rabbit mAb (#9664, Cell signaling), GAPDH (D16H11) Rabbit mAb (#5174, Cell signaling).

Wavelength screen assay. 1×10^4 SK-MEL-28 cells were seeded on 96-well plates and cultured in 37 ℃, 5% CO_2 for 12 h, followed by treatment with polycarcin V in as complete darkness as possible. 30 min later, the cells were irradiated by 365 nm, 400 nm, 450 nm, 500 nm, and 550 nm for 30 min respectively. With tin foil paper wrapping, the tested cells were cultured in darkness for 48 h, and the viability and IC_{50} was determined by celltiter-glo kit.

Cell fluorescence localization assay. 3000 HeLa cells were seeded on 96-well plate (black, transparent bottom) and cultured for 12 h. Cells were fixed with 200 μL 4% formaldehyde at room temperature for 20 min, washed twice with PBS buffer, treated with 200 μL 0.5% triton 100 for 20 min, and washed twice with 0.05% tween20. Then cells were blocked with 200 μL BSA at 37℃ for 1 h, followed by treatment with 20 μM polycarcin V or 50 μg/mL propidium iodide (PI) under visible light for 20 min. Washed with PBS three times and H_2O one time, and with the autofluorescent property of polycarcin V and PI, the fluorescence signals at 360 nm and 540 nm of excitation were observed by Opera.

Cell cycle analysis. The effect of polycarcin V on cell cycle was analyzed by flow

cytometry using propidium iodide (PI) DNA staining. HeLa cells were treated with DMSO or 1 nM polycarcin V for 24 h, and fixed in 70% ethanol at −20 ℃ for 24 h. Cells were incubated with 50 μg/mL PI and 20 μg/mL RNase A for 30 min at room temperature. Stained cells were analyzed in a flowcytometer (BD LSRFortessaTM Cell Analyzer).

Fluorescence studies. Calf thymus (CT) DNA was dissolved in 10 mM Tris-EDTA buffer at pH 7.1. The concentration of DNA was determined using nanodrop 2000 (ε_{260} = 6600 M^{-1} cm). 1.2 mg polycarcin V was prepared in 0.4 mL DMSO as stock, and diluted with 10 mM Tris-EDTA to the desired working concentrations. 0.39 μM polycarcin V was mixed with different concentrations of DNA under dark condition for 2 min, the DNA concentrations are 0 nM, 0.5 nM, 1 nM, 2.5 nM, 12.5 nM, 25 nM, 100 nM, 250 nM, 500 nM, 1 μM, 10 μM, 25 μM, 50 μM, 100 μM. Fluorescence spectra were recorded from 370 nm to 620 nm after an equilibration period of 2 min.

Circular dichroism spectrum assay. The annealed double strand DNA was prepared in different concentrations: 0 μM, 10 μM, 50 μM, 75 μM, 100 μM, 125 μM, 150 μM, 200 μM, and incubated with 1 mM polycarcin V under 365 nm light for 30 min. Circular dichroism spectrum was recorded from 200-500 nm. The DNA sequence is as follow:

5’-CCAAATAAAAGGAAGTGAAACCAAGCT-3’

3’-GGTTTATTTTCCTTCACTTTGGTTCTC-5’

[2+2] cycloadduct isolation. 20 mg calf-thymus DNA were dissolved in 20 mL Tris buffer (10 mM Trizma base, 1 mM Na, EDTA, pH8.0). Polycarcin V was dissolved in DMSO to a concentration of 1 mg/mL. 1 mL polycarcin V was incubated with 20 mL DNA in darkness for 30 min, and then irradiated by 365 nm light for 30 min. To precipitate DNA, 70 mL pre-cooled ethanol and 10 mL 3M NaAc were added to the mixture, and placed at −20℃ for 1 h. The samples were centrifuged at 4℃, 10000 rpm for 30 min, and the supernatant was discarded. [2+2] DNA adducts were isolated by hydrolyzing with 10 mL 0.1 N HCl at 100 ℃ for 2 h.

High-throughput sequencing. HeLa cells were treated with 10 μM probe **1**, followed by 365 nm light irradiation for 30 min, and continue to culture at 37℃, 5% CO_2 for 12 h. Utilizing DNA extraction kit (Tiangen, DP302-02), 50 μg genomic DNA was

extracted and clicked with $CuSO_4$ (10 mM), BTTAA (1mM), sodium ascorbate (100 mM), and excessive azide-biotin, at room temperature for 2–3 h in 200 μL volume. Genomic DNA was fragmented into 100–200 bp by ultrasonic disruption (Bioruptor Sonication Device). DNA fragments were precipitated by 70% pre-cooled ethanol and 0.3 M NaAc at −20℃ for 1–2 h, and incubated with streptavidin beads to enrich the probe **1** modified DNA fragments. After washing with PBS 6 times, the beads was heated to 95℃ for 3 min to release the modified DNA. The modified DNA samples were irradiated at 254 nm for 10 min (Stratalinker) to reverse polycarcin V probe-DNA crosslinks, followed by library preparation, massively parallel sequencing and analysis of sequencing data.

Profiling of DNA-binding proteins by SILAC-ABPP. SILIAC DMEM was supplemented with 10% dialyzed SILAC FBS and 1% penicillin-streptomycin, and 100 μg/mL of regular L-arginine-HCl and L-lysine-HCl (Sigma-Aldrich) or [$^{13}C_6$,$^{15}N_4$]L-arginine-HCl and [$^{13}C_6$,$^{15}N_2$]L-lysine-HCl (Cambridge Isotope Laboratory) were added to make the light or heavy media. MCF-7 cells were cultures in the light or heavy media and passaged at least seven times to verify full incorporation of the isotopically labeled amino acids. The light and heavy MCF-7 SILAC cells were treated with 20 μM negative probe **10** and 20 μM positive probe **9** for 30 min, followed by 450 nm light irradiation for 20 min to crosslink DNA, and 365 nm light irradiation for 20 min to crosslink the DNA-binding protein. The same number of light and heavy cells were digested by trypsin, and collected for nuclei extraction. With nuclei extraction kit (Solarbio, SN0020), the nuclei were prepared, and then lysed by RIPA buffer for 30 min on the ice and ultrasonic crushing for 2 min. After centrifugation (15000 rpm, 10 min), the light and heavy cell lysis were mixed in 1:1 ratio, and clicked with CuSO4 (10 mM), BTTAA (1mM), sodium ascorbate (100 mM), and excessive azide-biotin, at room temperature for 2–3 h. Subsequently, proteins were precipitated by 50% acetone at −20℃ for 1 h, and centrifuged at 4 ℃, 15000 rpm, 10 min. With 100 μL 1% SDS resuspension, the precipitated proteins diluted to 0.2% SDS/PBS and incubated with 150 μL streptavidin beads overnight at 4℃. The beads were washed with PBS 4 times,

0.2% SDS twice, 6 M urea once, and suspended in 500 μL 6M urea/PBS. After reduction with 10 mM dithiothreitol (DTT) at 37℃ for 30 min and alkylation with 20 mM iodoacetamide (IAA) at 35 ℃ for 30 min in dark, the beads were washed with 2 M urea and incubated with 200 μL 2 M urea, 1 mM $CaCl_2$ and 2 μg trypsin in PBS at 37 ℃ with agitation for 17 h. The beads heated in 95℃ for 5 min, and the supernatant was collected for freeze-drying. After desalting with C18 column, the samples were dissolved in 0.1% formic acid, and analyzed by LC-MS/MS.

Cells used for Comet Chip and Clonogenic Survival Assays. Sv40-transformed fibroblast cells XP2YO (XPF-deficient, GM08437) and XP2OS (XPA-deficient) were cultured in Dulbecco's Modified Eagle's Medium (DMEM, Invitrogen) supplemented with 10% fetal calf serum and 2mM L-glutamine, 100U/ml penicillin, and 0.1mg/ml streptomycin at 37ºC in the presence of 5% CO_2. Cells complemented with XPF-WT or the catalytically inactive XPF-D687A and XPA-WT had been generated by lentiviral transduction[45].

CometChip Assays. The high-throughput alkaline comet assay (CometChip) was performed as described[40] with minor modifications. Cells were treated with 2.5 mM thymidine (Sigma) for 24 hours to arrest the cell cycle at G1 phase. Cells were then collected and resuspended in complete media and allowed to settle in microwells of a 96-well comet chip platform (Trevigen, 30 micron cat# 4260-096-01). Cells were treated with 2 nM of Polycarcin V ± 10 mM Cytarabine / 400mM Hydroxy urea for 2 h and then irradiated with 2 KJ/m^2 UV-A (365nm CL-1000 UVP crosslinker). Cells were subsequently incubated in complete media with 2.5 mM thymidine to keep the cells in G1 for 2 h at 37 °C. Medium was then replenished and cells incubated for another 0, 2 or 4 hours. After indicated time points, the chip was overlaid with 6 ml LM Agarose (Trevigen, cat# 4250-500-02), repair was stopped, cells lysed by incubating the chips in lysis solution (Trevigen, cat# 4250-500-01) for 1h at 4°C. The CometChip was equilibrated twice in alkaline electrophoresis buffer (200 mM NaOH/1 mM EDTA/0.1% Triton X-100) for 20 min at 4°C, and subjected to electrophoresis for 40 minutes at 22 V at 4°C. The chips were neutralized with 0.4 M Tris-HCl, pH 7.4 and equilibrated with 20 mM Tris-HCl, pH 7.4. Following overnight staining at 4°C in 100

ml 0.2X SYBR Gold (Invitrogen) images were captured on the fluorescence microscope, and comet tail lengths were analyzed using Comet Analysis Software (Trevigen, cat# 4260-000-CS). At least 100 cells per well were analyzed and each experiment was carried out in duplicate well.

Clonogenic Survival Assays. Exponentially growing cells were plated in duplicate in 6-cm dishes at 1500 cells/plate. After allowing the cells to adhere overnight, they were treated with medium containing Polycarcin V (0.1, 0.3, 1, 3 or 10nM) and UV (2kJ/m^2 UV-A; 365nm CL-1000 UVP crosslinker) and then incubated for 2 h at 37 °C. The drug was removed, fresh medium without Polycarcin V added and cells allowed to grow for 12 days. The cultures were fixed and stained with 2% of methylene blue (Sigma). Colonies (defined as ⩾50 cells) were counted. The data were plotted as the number of colonies that grew from the treated relative to untreated cells. Values ± S.E. were determined from two independent experiments.

IMQ-induced psoriasis-like skin disorder in mice. All animals were obtained from company (Charles River/Wei Tong Li Hua, China) and acclimated for about one week before experimental treatment. All the animal experiments were conducted in the barrier facility of laboratory animal center, and approved by the institutional animal use and care committee.

Female BALB/c (8weeks) backs were shaved 1 day before treatment with an electric clipper, the remaining short hair were removed with depilation cream and then cleaned with warm water. Animals were treated with daily topical application of imiquimod (IMQ) cream (62.5 mg/mouse/day) on back for 7 consecutive days after removing back hair one day in advance, petrolatum cream as health control. 2 h before the cream application, 200 uL of vehicle (60% ethanol), or different concentration of compounds tincture (5, 20 or 100 μg/mL for 1, 5 or 20 μg/mouse) were topically administered to the fixed hair removal area of ~1.5cm^2. About 20 minutes after tincture treatment, 365nm or blue light (440-470nm, about 40mM/cm^2) irradiation were performed at a distance about 15cm for designed time from the dorsal animal back skin. Back skin of all animals was daily photographed. The scales and erythema scores were also monitored daily by an independent investigator, following clinical psoriasis area and

severity index (PASI): erythema and scales are calculated respectively on a scale from 0-4: 0, none; 1, slight; 2, moderate; 3, marked and 4, highly marked. After the last treatment, fasted overnight, mice were killed by cervical dislocation, ~1cm^2 biopsies were taken from the treated dorsal skin of the same area on all animals for subsequent formalin fixation and paraffin-embedded histological sections.

IV) Chemical synthesis

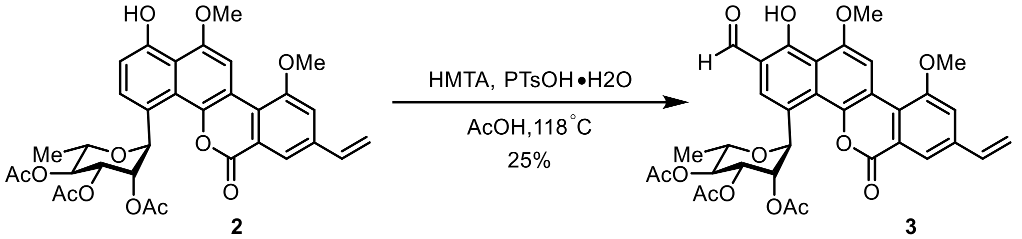

Compound 3. 2 (61.2 mg, 0.0986 mmol) was dissolved in acetic acid (3ml) and treated with HMTA(15 mg, 0.0986 mmol) and PTsOH·H_2O (15 mg, 0.0789 mmol). The reaction was refluxed at 118 °C under argon for 5 h. The reaction mixture was then cooled to room temperature and diluted with CH_2Cl_2. Saturated sodium carbonate solution was slowly added until the aqueous layer became basic. The organic layer was separated, dried over Na_2SO_4, and concentrated *in vacuo*. The residue was purified by silica gel column chromatography (DCM/MeOH=100/1) to afford **3** (15.9 mg, 25%) as a yellow solid.

Mp 210–217°C;

^{1}H NMR (400 MHz, $CDCl_3$) δ 10.53 (s, 1H), 8.54 (s, 1H), 8.23 (s, 1H), 8.19 (d, *J* = 1.6 Hz, 1H), 7.37 (d, *J* = 1.5 Hz, 1H), 6.80 (dd, *J* = 17.5, 10.9 Hz, 1H), 6.24 (s, 1H), 5.97 (d, *J* = 17.5 Hz, 1H), 5.89 (d, *J* = 3.3 Hz, 1H), 5.68 (dd, *J* = 10.0, 3.3 Hz, 1H), 5.49 (d, *J* = 10.9 Hz, 1H), 5.23 (ddd, *J* = 13.3, 10.0, 7.6 Hz, 2H), 4.15 (s, 3H), 4.12 (s, 3H), 2.11 (d, *J* = 3.3 Hz, 3H), 1.95 (s, 3H), 1.86 (s, 3H), 1.40 (d, *J* = 6.1 Hz, 3H).

^{13}C NMR (125 MHz, $CDCl_3$) δ 190.2, 170.4, 170.0, 161.2, 160.2,159.5, 157.9, 153.9, 142.0, 140.0, 135.2, 126.5, 126.1, 124.5, 123.2, 122.8, 120.7, 118.0, 117.7, 117.5, 116.0, 114.7, 104.5, 76. 6, 75.0, 72.6, 72.1, 71.4, 56.7, 56.6, 21.1, 20.8, 20.7, 18.2.

HRMS (ESI) $[M + H]^+$ calculated for $C_{34}H_{33}O_{13}$: 649.1921, found: 649.1928;

TLC: R_f = 0.47 (DCM/MeOH=100/1).

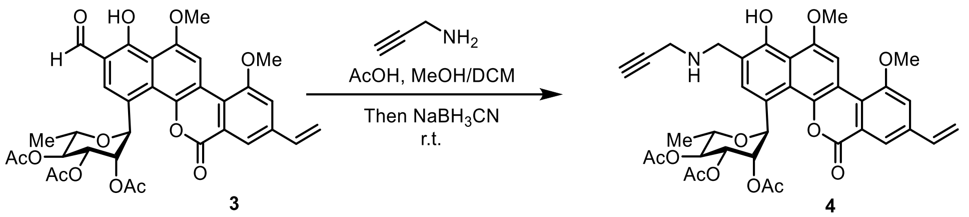

Compound 4. 3 (9.0 mg, 0.0139 mmol) was dissolved in MeOH/DCM (1ml/1ml) and treated with propargylamine (2 μL, 0.0278 mmol) and acetic acid (0.8 μL, 0.0139 mmol). The reaction was stirred at room temperature under argon for 6 h. Then, $NaBH_3CN$ (9.0 mg, 0.139 mmol) was added and the mixture was stirred for 2 h. Then 3 ml water was added to the mixture. The organic layer was separated, dried over Na_2SO_4, and concentrated *in vacuo* to afford **4** as a yellow solid used directly in next step.

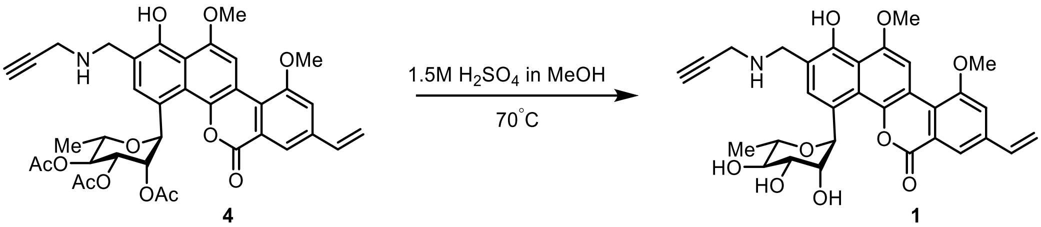

Probe 1. To a suspension of **4** (10 mg, 0.0140 mmol) in 1.5 mL of MeOH was added 1.5 mL of 3.0 M H_2SO_4 in MeOH. The resulting reaction solution was stirred at 70 ºC for 7 h and then cooled to r.t. The reaction was diluted with water (5 mL), and extracted with $CHCl_3$/*i*-PrOH (3/1) three times. The organic phases were washed with saturated aqueous $NaHCO_3$ and then dried over anhydrous Na_2SO_4, filtered and concentrated *in vacuo*. The residue was purified by flash chromatography on silica gel (CH_2Cl_2/MeOH = 90/10) to give **1** (4.9 mg, 63% for 2 steps) as a yellow solid.

Mp 164–170 ºC;

^{1}H NMR (400 MHz, MeOH-d4) δ 8.24 (s, 1H), 7.82 (s, 1H), 7.69 (s, 1H), 7.25 (s, 1H), 6.78 (dd, *J* = 17.5, 10.9 Hz, 1H), 5.97 (d, *J* = 17.6 Hz, 1H), 5.74 (s, 1H), 5.47 (d, *J* = 10.9 Hz, 1H), 4.16 (d, *J* = 6.7 Hz, 1H), 4.14 (s, 3H), 4.04 (dd, *J* = 11.2, 3.1 Hz, 2H), 4.00 (s, 3H), 3.83 (t, *J* = 2.5 Hz, 2H), 3.47 (t, *J* = 9.3 Hz, 2H), 3.08 (t, *J* = 2.4 Hz, 1H), 1.53 (t, *J* = 6.0 Hz, 3H).

^{13}C NMR (126 MHz, MeOH-*d4*) δ 161.2, 158.8, 153.4, 153.2, 143.9, 140.6, 136.5,

131.6, 131.1, 128.6, 126.3, 123.8, 123.2, 120.4, 117.5, 116.1, 115.5, 115.2, 103.5, 78.9, 78.2, 77.6, 76.1, 74.7, 73.8, 57.0, 56.9, 55.2, 47.7, 30.8, 18.7.

HRMS (ESI) $[M + H]^+$ calculated for $C_{31}H_{32}NO_9$: 562.2077, found: 562.2074;

TLC: R_f = 0.37 (DCM/MeOH=90/10).

[2+2] Cycloaddition Product Preparation and Isolation: Polycarcin V was prepared by total synthesis. Samples of calf-thymus DNA (Sigma), 20 mg each, were dissolved in 20 mL of Tris buffer (10 mM TRIZMA base, 1 mM Na, EDTA, pH 8.0). Polycarcin V was dissolved in DMSO to a concentration of 1 mg/mL. In dark condition, 1 mL of the gilvocarcin V solution was added to each sample of DNA solution. After vigorous shaking, the samples were placed in a clear fused quartz glass tube. The samples were photolyzed for 30 min. After photolysis, 5 mL of saturated NaCl solution was added, and the mixture was poured into 95mL of cold ethanol. The resulting precipitate was wound onto a glass rod and pressed to remove additional ethanol. The precipitated DNA pellets were a yellow-orange color. DNA adducts were isolated by hydrolyzing the combined DNA pellets from 16 photolysis experiments with 10 mL of 0.1 N HCI at 100 ℃ for 2 h. This solution was filtered through Waters C-18 Sep-Pak cartridges. The cartridges were rinsed with water and increasing percentages of methanol in water. Adducts containing the polycarcin V chromophore eluted with 60−70% methanol in water. The major [2+2] cycloaddition product was characterized by NMR analysis.

^{1}H NMR (400 MHz, MeOH-*d4*) δ 8.44 (s, 1H), 7.87 (d, *J* = 8.3 Hz, 1H), 7.76 (s, 1H), 7.29 (s, 1H), 6.93 (d, *J* = 8.4 Hz, 1H), 5.93 (s, 1H), 4.58 (s, 1H), 4.23 (d, *J* = 3.1 Hz, 1H), 4.13 (s, 3H), 4.12 (s, 3H), 3.49 (d, *J* = 9.4 Hz, 2H), 2.79-2.69 (m, 1H), 2.60-2.52 (m, 1H), 2.08-2.00 (m, 1H), 1.63 (s, 3H), 1.46 (d, *J* = 6.0 Hz, 3H).

UPLC-MS (ESI) $[M + H]^+$ calculated for $C_{32}H_{33}N_2O_{11}$: 621.2084, found: 621.96;

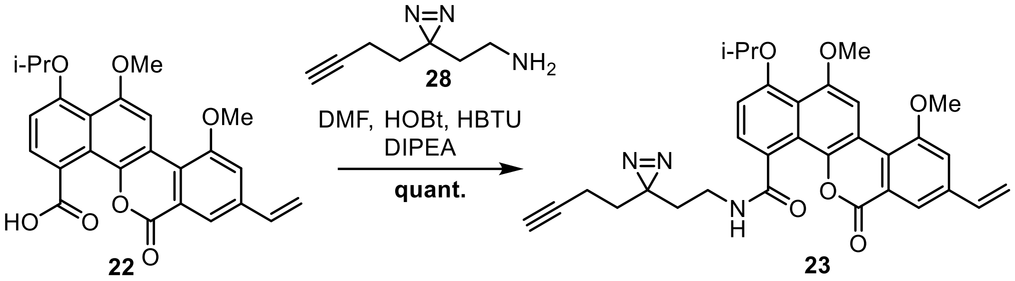

Acid **22**[1] and amine **28**[4] were prepared according to the literature. To a stirring solution of acid **22** (40 mg, 0.092 mmol) in dry DMF (8 mL) was successively added N-hydroxybenzotriazole (12.4 mg, 0.092 mmol), diisopropylethylamine (50 μL, 0.27 mmol), and HBTU (60 mg, 0. 158 mmol) at 25 °C. After stirring at this temperature for 3 h, amine **23** (37 mg, 0.276 mmol) was added to the reaction mixture then the mixture was stirred under nitrogen for 12 h and concentrated *in vacuo* to afford a crude yellow solid, which was purified by silica gel column chromatography (CH_2Cl_2 /MeOH = 100 /1) to afford **7** (50 mg, quant.) as a yellow solid.

Data of **23**: **^{1}H NMR** (500 MHz, $CDCl_3$) δ 8.38 (s, 1H), 8.07 (d, J = 1.6 Hz, 1H), 7.42 (d, J = 8.0 Hz, 1H), 7.25 (d, J = 1.3 Hz, 1H), 6.91 (d, J = 8.0 Hz, 1H), 6.79 (dd, J = 17.5, 10.9 Hz, 1H), 6.18 (t, J = 5.5 Hz, 1H), 5.93 (d, J = 17.5 Hz, 1H), 5.44 (d, J = 10.9 Hz, 1H), 4.61 (dq, J = 12.1, 6.0 Hz, 1H), 4.06 (s, 3H), 3.99 (s, 3H), 3.55 (q, J = 6.7 Hz, 2H), 2.08 (td, J = 7.4, 2.6 Hz, 2H), 2.03 (t, J = 6.8 Hz, 2H), 1.91 (t, J = 2.6 Hz, 1H), 1.78 (t, J = 7.4 Hz, 2H), 1.44 (d, J = 6.0 Hz, 6H).

^{13}C NMR (126 MHz, $CDCl_3$) δ 171.7, 159.9, 157.4, 155.4, 152.9, 140.4, 138.7, 135.4, 127.4, 126.7, 123.5, 123.4, 123.0, 120.3, 119.8, 116.4, 114.3, 113.8, 112.4, 106.2, 82.9, 73.0, 69.2, 57.1, 56.2, 35.3, 32.3, 32.2, 27.3, 22.0, 13.3.;

IR (film, cm^{-1}) 3357, 2955, 2927, 2854, 1724, 1654, 1589, 1457, 1380, 1261, 1106, 804;

HRMS(ESI) $[M + H]^+$ calculated for $C_{32}H_{32}N_3O_6$: 554.2286, found: 554.2293;

TLC: Rf = 0.5 (CH_2Cl_2/MeOH = 20/1).

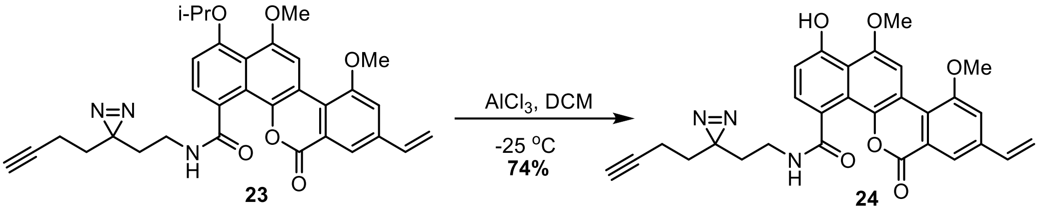

To a stirred solution of **23**(25.0 mg, 0.045 mmol) in CH_2Cl_2 (6 mL) at −25 °C was added $AlCl_3$ (30 mg, 0.225 mmol). After stirring at this temperature for 2 h, the excess reagents were quenched by water (6 mL), the resulting mixture was extracted with CH_2Cl_2 (6 mL × 3), washed with brine (20 mL), the combined organic phases were dried over anhydrous Na_2SO_4, concentrated *in vacuo* to afford a crude yellow solid, which was purified by flash chromatography (CH_2Cl_2 /MeOH = 100 /1) to afford **24** as a yellow solid (17.4 mg, 74%).

Data of **24**: ^{1}**H NMR** (400 MHz, $CDCl_3$) δ 9.65 (s, 1H), 8.29 (s, 1H), 7.99 (d, J = 1.5 Hz, 1H), 7.45 (d, J = 8.0 Hz, 1H), 7.25 (s, 1H), 6.92 (d, J = 8.0 Hz, 1H), 6.73 (dd, J = 17.6, 10.9 Hz, 1H), 5.98 – 5.87 (m, 2H), 5.43 (d, J = 10.9 Hz, 1H), 4.08 (s, 3H), 4.06 (s, 3H), 3.49 (q, J = 6.6 Hz, 2H), 2.05 (td, J = 7.4, 2.7 Hz, 2H), 2.01 (t, J = 6.7 Hz, 2H), 1.89 (t, J = 2.6 Hz, 1H), 1.75 (t, J = 7.4 Hz, 2H).

^{13}**C NMR** (150 MHz, $CDCl_3$) δ 171.3, 159.7, 157.4, 155.3, 151.8, 141.0, 139.0, 135.2, 128.9, 125.0, 123.4, 123.0, 122.4, 120.4, 116.7, 115.0, 114.0, 113.9, 111.7, 102.8, 82.9, 69.2, 56.3, 35.1, 32.1, 32.0, 29.7, 27.2, 13.3.

IR (film, cm^{-1}) 3364, 3301, 2923, 2854, 1723, 1651, 1616, 1448, 1380, 1245, 1129, 783;

HRMS(ESI) $[M + H]^+$ calculated for $C_{29}H_{26}N_3O_6$: 512.1816, found: 512.1824;

TLC: Rf = 0.5 (CH_2Cl_2/MeOH = 20/1).

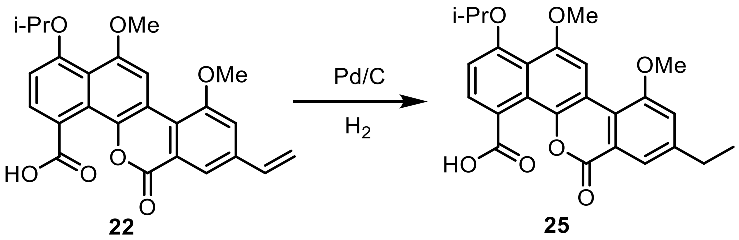

To a stirred solution of **22** (20.0 mg, 0.046 mmol) in ethyl acetate (130 mL) and MeOH (30 mL) was added 10% Pd/C (20 mg). The resulting mixture was degassed at −78 ºC and backfilled with H_2 three times and equipped with an H_2-filled balloon. Then the reaction was stirred at 25 ºC for 12 h followed by concentration *in vacuo* to afford a yellow solid which was used directly in the next step.

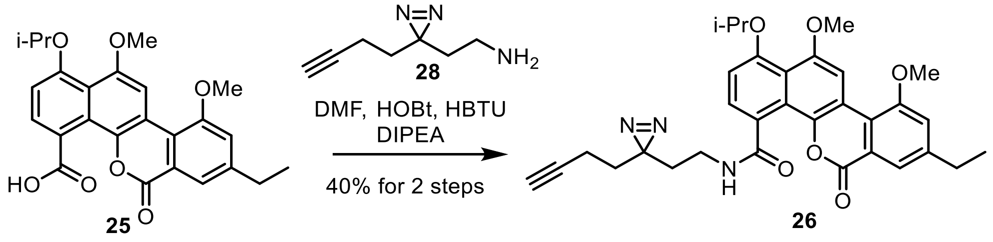

To a stirring solution of crude **25** in dry DMF (4 mL) was successively added N-hydroxybenzotriazole (6.2 mg, 0.046 mmol), diisopropylethylamine (25 µL, 0.14 mmol), and HBTU (35 mg, 0. 192mmol) at 25 ºC. After stirring at this temperature for 3 h, amine **28** (19 mg, 0.14 mmol) was added to the reaction mixture then the mixture was stirred under nitrogen for 12 h and concentrated *in vacuo* to afford a crude yellow solid, which was purified by silica gel column chromatography (CH_2Cl_2 /MeOH = 100 /1) to afford **26** (10 mg, 40% for 2 steps) as a yellow solid.

Data of **26**: **^{1}H NMR** (500 MHz, $CDCl_3$) δ 8.43 (s, 1H), 7.91 (d, J = 1.5 Hz, 1H), 7.44 (d, J = 8.0 Hz, 1H), 7.12 (d, J = 1.3 Hz, 1H), 6.93 (d, J = 8.0 Hz, 1H), 6.05 (t, J = 5.7 Hz, 1H), 4.62 (dq, J = 12.1, 6.0 Hz, 1H), 4.06 (s, 3H), 3.98 (s, 3H), 3.54 (q, J = 6.7 Hz, 2H), 2.78 (q, J = 7.6 Hz, 2H), 2.07 (td, J = 7.4, 2.6 Hz, 2H), 2.02 (t, J = 6.8 Hz, 2H), 1.89 (t, J = 2.6 Hz, 1H), 1.77 (t, J = 7.4 Hz, 2H), 1.44 (d, J = 6.0 Hz, 6H), 1.33 (t, J = 7.6 Hz, 3H).

^{13}C NMR (126 MHz, $CDCl_3$) δ 171.7, 160.1, 157.3, 155.5, 152.9, 146.2, 140.1, 127.4, 126.7, 123.3, 123.1, 121.8, 121.3, 119.7, 116.9, 114.6, 112.1, 106.5, 82.9, 72.9, 69.1, 57.2, 56.2, 35.2, 32.2, 32.2, 28.9, 27.3, 22.0, 15.1, 13.3.

IR (film, cm^{-1}) 3262, 2973, 2933, 1721, 1627, 1588, 1535, 1382, 1249, 1139, 1057, 1010, 917, 848, 785;

HRMS(ESI) $[M + H]^+$ calculated for $C_{32}H_{34}N_3O_6$ 556.2442, found: 556.2446;

TLC: Rf = 0.5 (CH_2Cl_2/MeOH = 20/1).

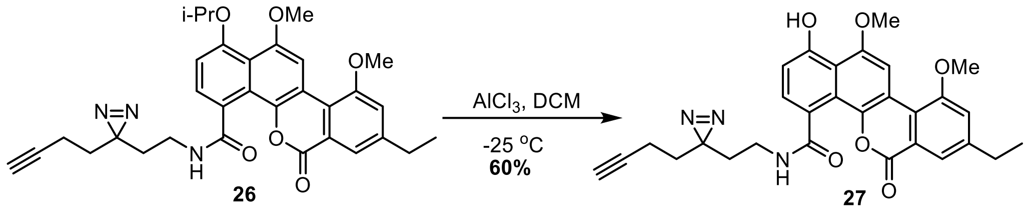

To a stirred solution of **26** (9.0 mg, 0.016 mmol) in CH_2Cl_2 (2 mL) at −25 °C was added $AlCl_3$ (11 mg, 0.08 mmol). After stirring at this temperature for 2 h, the excess reagents were quenched by water (2 mL), the resulting mixture was extracted with CH_2Cl_2 (2 mL x 3), washed with brine (6 mL), the combined organic phases were dried over anhydrous Na_2SO_4, concentrated *in vacuo* to afford a crude yellow solid, which was purified by silica gel preparative TLC (CH_2Cl_2 /MeOH = 40 /1) to afford **27** as a yellow solid (4.9 mg, 60%).

Data of **27:** ^{1}**H NMR** (600 MHz, $CDCl_3$) δ 9.70 (s, 1H), 8.47 (s, 1H), 7.95–7.91 (m, 1H), 7.48 (d, J = 7.9 Hz, 1H), 7.20 –7.18 (m, 1H), 6.95 (d, J = 7.9 Hz, 1H), 5.89 (t, J = 5.7 Hz, 1H), 4.15 (s, 3H), 4.09 (s, 3H), 3.51 (q, J = 6.5 Hz, 2H), 2.80 (q, J = 7.6 Hz, 2H), 2.05 (td, J = 7.4, 2.6 Hz, 2H), 2.02 (t, J = 6.7 Hz, 2H), 1.88 (t, J = 2.6 Hz, 1H), 1.75 (t, J = 7.4 Hz, 2H), 1.34 (t, J = 7.6 Hz, 3H).

^{13}**C NMR** (150 MHz, $CDCl_3$) δ 171.4, 159.9, 157.2, 155.3, 151.9, 146.6, 140.8, 128.9, 125.0, 123.3, 122.5, 121.6, 121.5, 117.1, 114.9, 114.2, 111.5, 103.1, 82.9, 69.2, 56.4, 56.3, 35.1, 32.2, 32.1, 29.0, 27.3, 15.1, 13.3.

IR (film, cm^{-1}) 3227, 3060, 2920, 2850, 1716, 1614, 1585, 1424, 1381, 1366, 1300, 1133, 1054, 784;

HRMS(ESI) $[M + H]^+$ calculated for $C_{29}H_{28}N_3O_6$: 514.1973, found: 514.1971;

TLC: Rf = 0.5 (CH_2Cl_2/MeOH = 20/1).

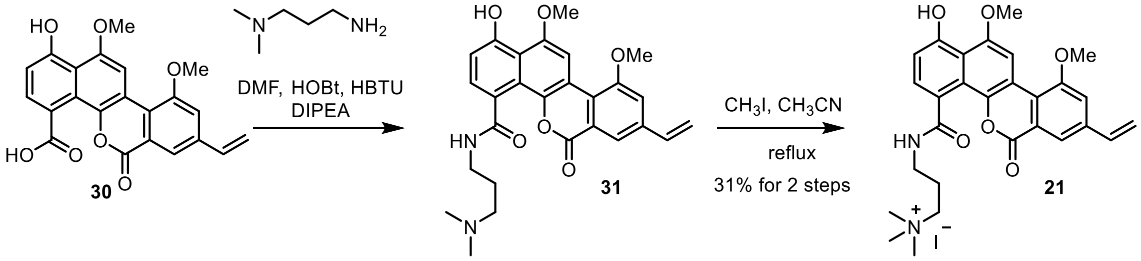

To a stirring solution of **30** (10.0 mg, 0.023 mmol) in dry DMF (1 mL) was successively added N-hydroxybenzotriazole (3.1 mg, 0.023 mmol), diisopropylethylamine (19 µL, 0.115 mmol), and HBTU (9.4 mg, 0.028 mmol) at 25 °C. After stirring at this temperature for 10 min, DMAPA (15 µL, 0.115 mmol) was added to the reaction mixture then the mixture was stirred under nitrogen for 12 h and concentrated *in vacuo* to afford a crude yellow solid, which was used directly in next step.

Crude **31** was taken in dry acetonitrile (2 mL) and methyl iodide (28 µl, 0.44 mm0l) was added to it and then refluxed for 24 h. Then the reaction was allowed to come to room temperature and the solvent was evaporated. The crude product was purified by PTLC (CH_2C1_2/MeOH = 90/10) to afford **21** (3.5 mg, 31% for 2 steps) as a yellow solid.
^{1}H NMR (400 MHz, CD_3CN) δ 8.41 (s, 1H), 7.89 (s, 1H), 7.52 (s, 1H), 7.38 (d, J = 8.0 Hz, 1H), 6.85 – 6.80 (m, 2H), 6.03 (d, J = 17.5 Hz, 1H), 5.47 (d, J = 10.8 Hz, 1H), 4.12-4.11 (m, 6H), 3.50 (t, J = 5.6 Hz, 3H), 3.26 (t, J = 5.6 Hz,2H), 2.85 (s, 9H), 2.12-2.08 (m, 2H).
HRMS(ESI) $[M - I]^+$ calculated for $C_{28}H_{31}N_2O_6$: 491.2177, found: 491.2171;
TLC: Rf = 0.25 (DCM /MeOH = 9/1).

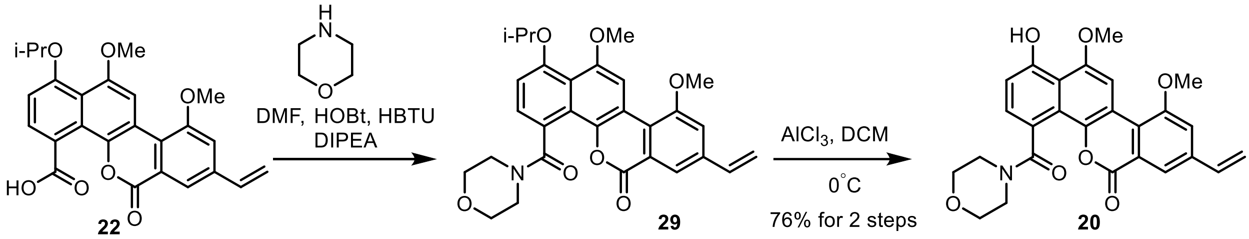

Compound **29**. To a stirring solution of **22**(20.0 mg, 0.046 mmol) in dry DMF (4 mL) was successively added N-hydroxybenzotriazole (6.2 mg, 0.046 mmol), diisopropylethylamine (25 µL, 0.14 mmol), and HBTU (35 mg, 0. 192mmol) at 25 °C. After stirring at this temperature for 3 h, morpholine (20 µL, 0.23 mmol) was added to the reaction mixture then the mixture was stirred under nitrogen for 12 h and concentrated *in vacuo* to afford a crude yellow solid, which was used directly in next step

Compound **20.** To a stirred solution of **29** in CH_2Cl_2 (2 mL) at 0 °C was added $AlCl_3$ (30.6 mg, 0.23 mmol). After stirring at this temperature for 2 h, the excess reagents were quenched by water (5 mL), the resulting mixture was extracted with CH_2Cl_2 (10 mL x 3), washed with brine (6 mL), the combined organic phases were dried over anhydrous Na_2SO_4, concentrated *in vacuo* to afford a crude yellow solid, which was purified by silica gel preparative TLC (PE /EtOAc = 4 /1) to afford **20** as a yellow solid (16.1 mg, 76% for 2 steps).

^{1}H NMR (500 MHz, $CDCl_3$) δ 8.43 (s, 1H), 8.08 (d, *J* = 1.3 Hz, 1H), 7.36 (d, *J* = 8.0 Hz, 1H), 7.35 (s, 1H), 7.01 (d, *J* = 8.0 Hz, 1H), 6.78 (dd, *J* = 17.5, 10.9 Hz, 1H), 5.94 (d, *J* = 17.5 Hz, 1H), 5.46 (d, *J* = 10.8 Hz, 1H), 4.22 – 4.15 (m, 2H), 4.13 (s, 3H), 4.11 (s, 3H), 4.02 (dd, *J* = 12.8, 5.2 Hz, 1H), 3.83 – 3.77 (m, 1H), 3.66 – 3.60 (m, 1H), 3.54 (d, *J* = 4.8 Hz, 1H), 3.28 (d, *J* = 4.2 Hz, 2H).

^{13}C NMR (126 MHz, $CDCl_3$) δ 172.2, 159.8, 157.5, 155.4, 152.1, 140.8, 139.4, 135.2, 128.3, 123.3, 123.0, 122.4, 121.9, 120.5, 116.9, 115.1, 114.3, 112.3, 103.1, 100.0, 66.24, 66.08, 56.40, 47.60.

HRMS(ESI) $[M + H]^+$ calculated for $C_{26}H_{24}NO_7$: 462.1553, found: 462.1548;

TLC: Rf = 0.4 (PE /EtOAc = 2/1).

IV) NMR Spectra

^{1}H NMR and UPLC-MS of [2+2] Cycloaddition Product

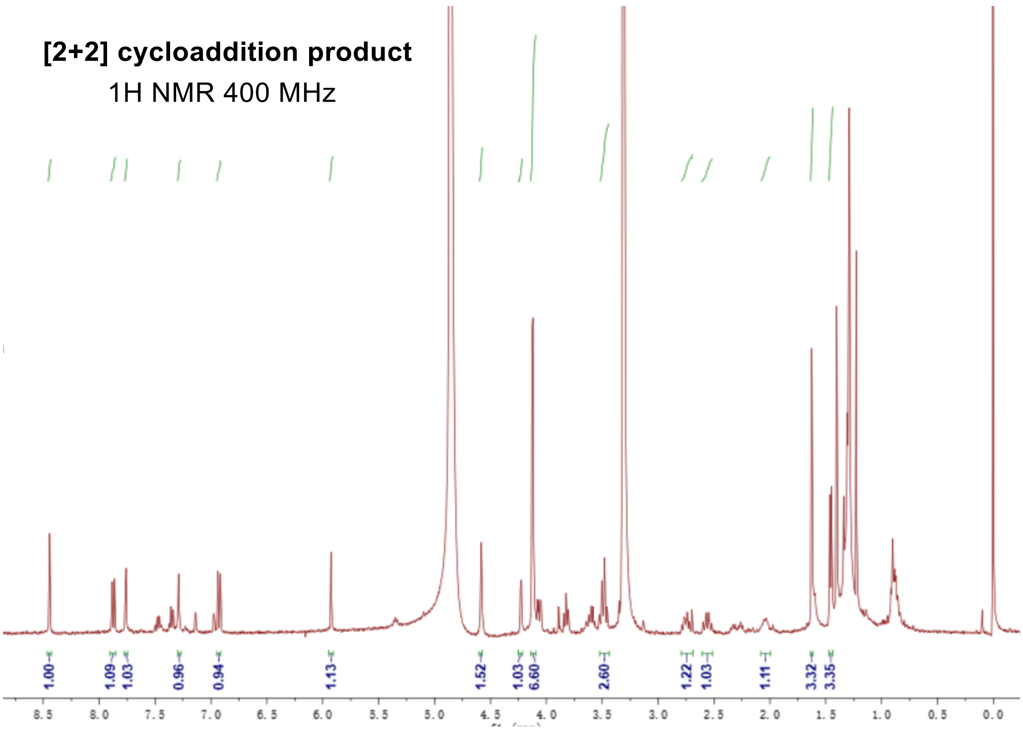
[2+2] cycloaddition product
1H NMR 400 MHz

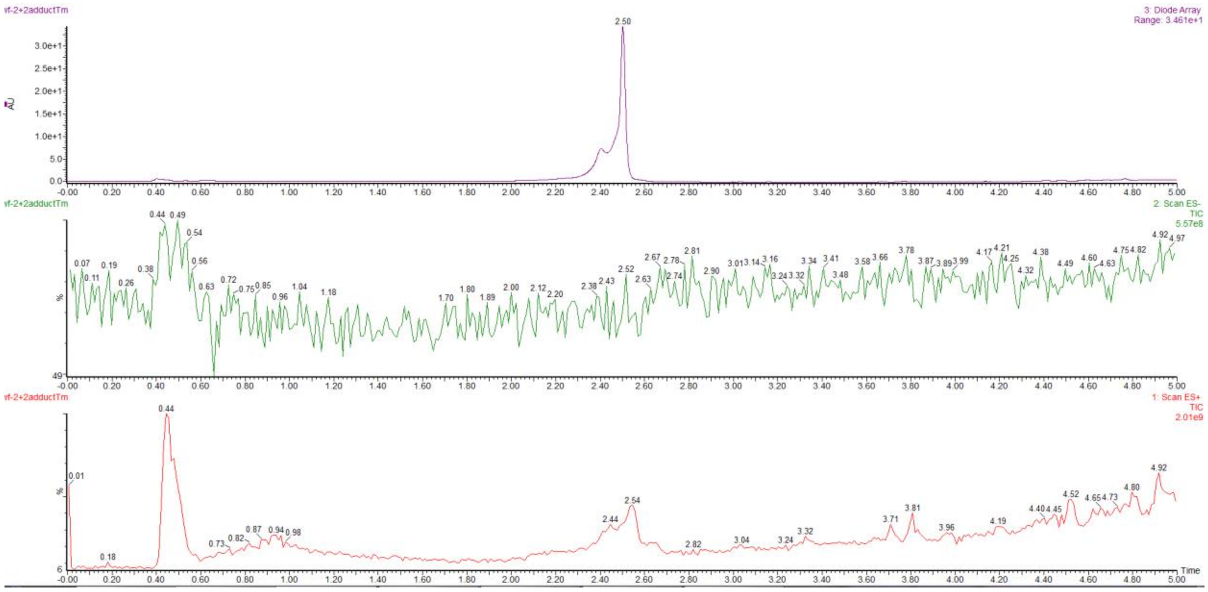
3: Diode Array
Range: 3.461e+1
2: Scan ES-
TIC
5.57e8
1: Scan ES+
TIC
2.01e9
Time

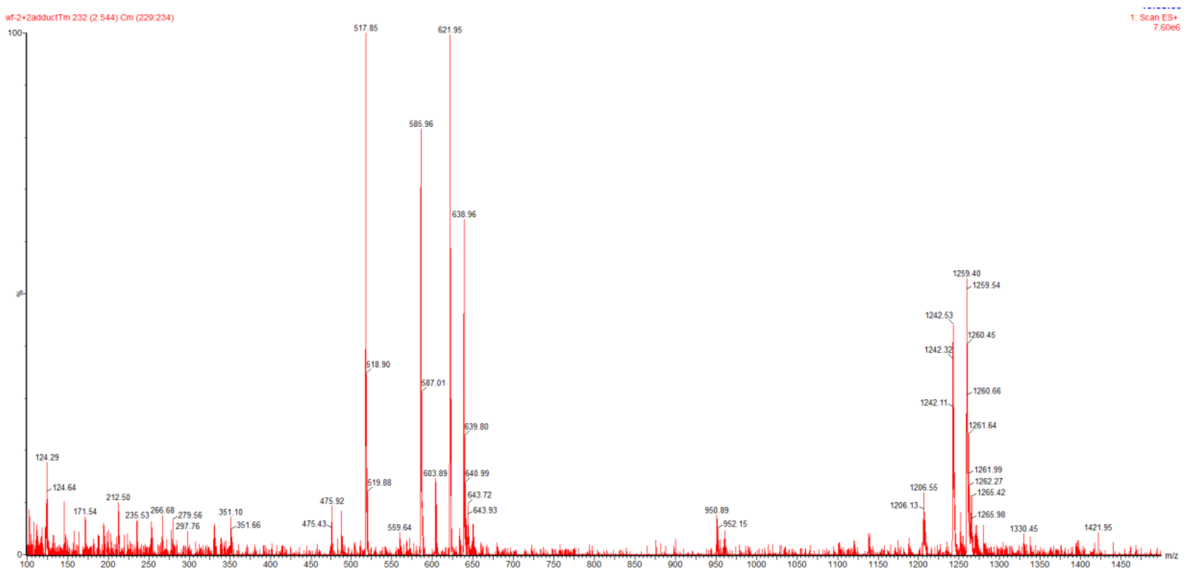
wf-2+2adductTm 232 (2.544) Cm (229:234)
1: Scan ES+
7.60e6
100
%
0
517.85
621.95
585.96
638.96
518.90
587.01
639.80
519.88
603.89
640.99
643.72
643.93
1259.40
1259.54
1242.53
1260.45
1242.32
1260.66
1242.11
1261.64
1261.99
1262.27
1265.42
1265.98
1206.55
1206.13
1330.45
1421.95
950.89
952.15
124.29
124.64
171.54
212.50
235.53
266.68
279.56
297.76
351.10
351.66
475.92
475.43
559.64
m/z

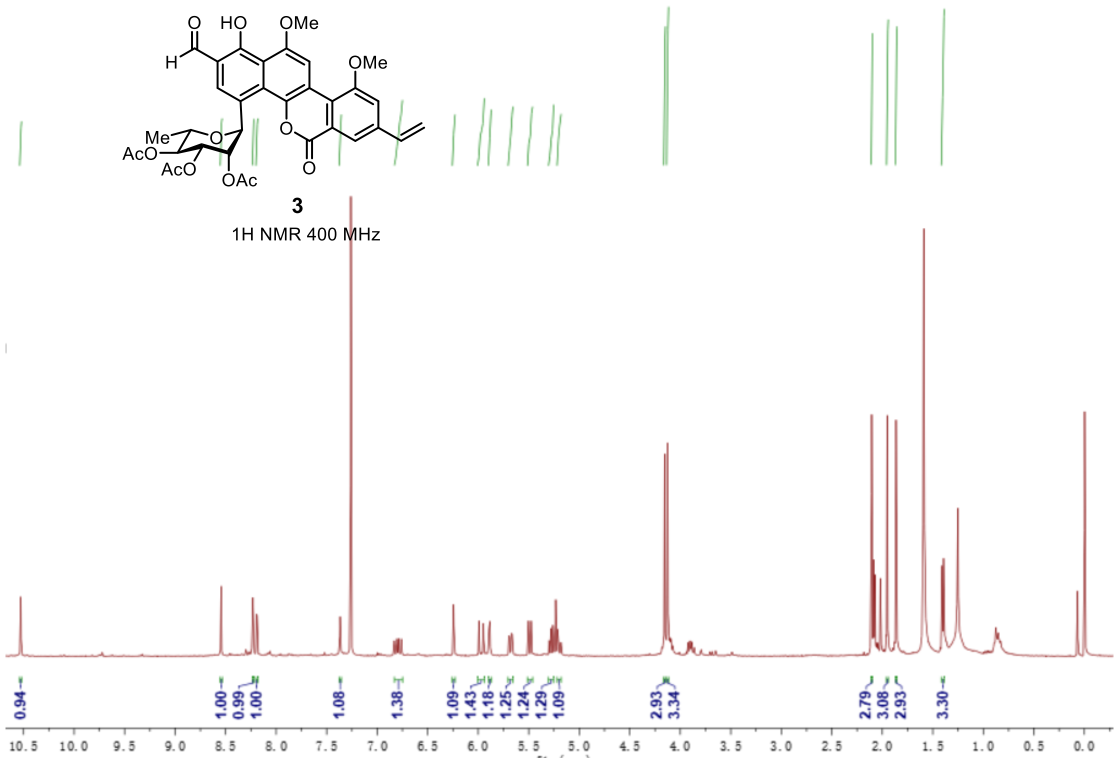
O HO OMe
H
OMe
Me O
AcO
AcO
OAc
O
O
3
1H NMR 400 MHz
0.94
1.00
0.99
1.00
1.08
1.38
1.09
1.43
1.18
1.25
1.24
1.29
1.09
2.93
3.34
2.79
3.08
2.93
3.30
10.5 10.0 9.5 9.0 8.5 8.0 7.5 7.0 6.5 6.0 5.5 5.0 4.5 4.0 3.5 3.0 2.5 2.0 1.5 1.0 0.5 0.0

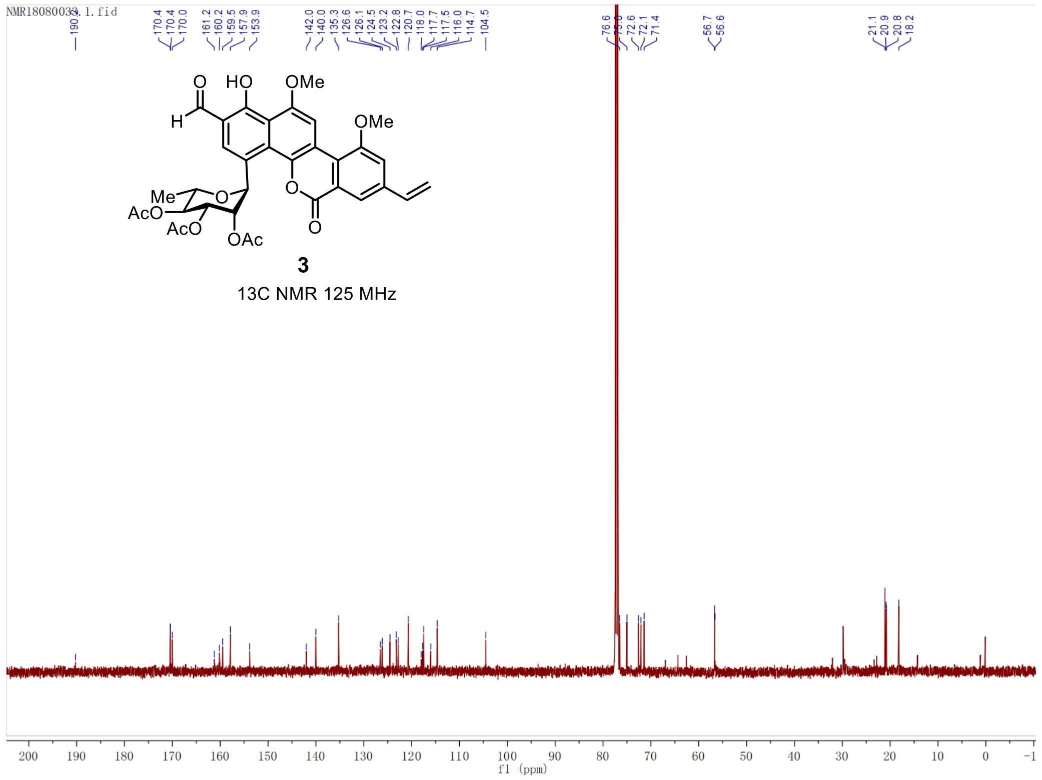
170.4
170.4
170.0
161.2
160.2
159.5
157.9
153.9
142.0
140.0
135.3
126.6
126.1
124.5
123.2
122.8
120.7
118.0
117.7
117.5
116.0
114.7
104.5
76.6
72.6
72.1
71.4
56.7
56.6
21.1
20.9
20.8
18.2
O HO OMe
H
OMe
Me O
AcO
AcO
OAc
O
O
3
13C NMR 125 MHz
200 190 180 170 160 150 140 130 120 110 100 90 80 70 60 50 40 30 20 10 0 -1
f1 (ppm)

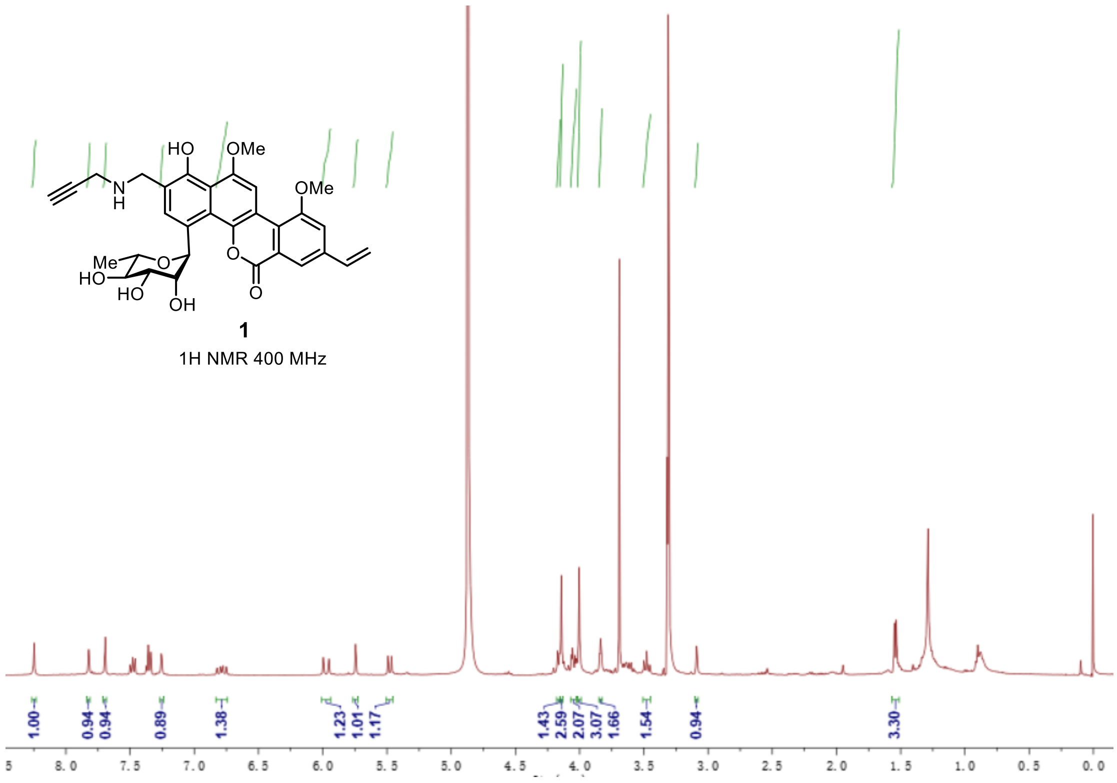
HO
OMe
OMe
N
H
Me
O
HO
HO
OH
O
O
1
1H NMR 400 MHz

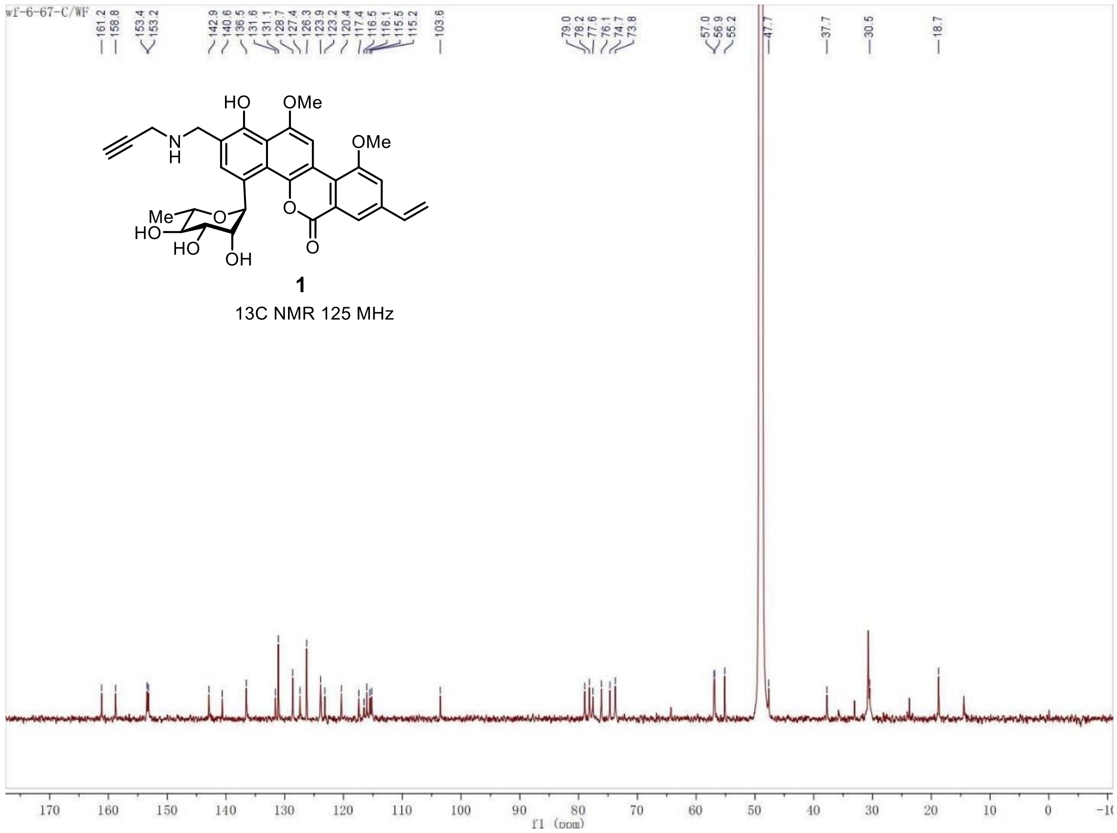
wf-6-67-C/WF
HO
OMe
OMe
N
H
Me
O
HO
HO
OH
O
O
1
13C NMR 125 MHz
f1 (ppm)

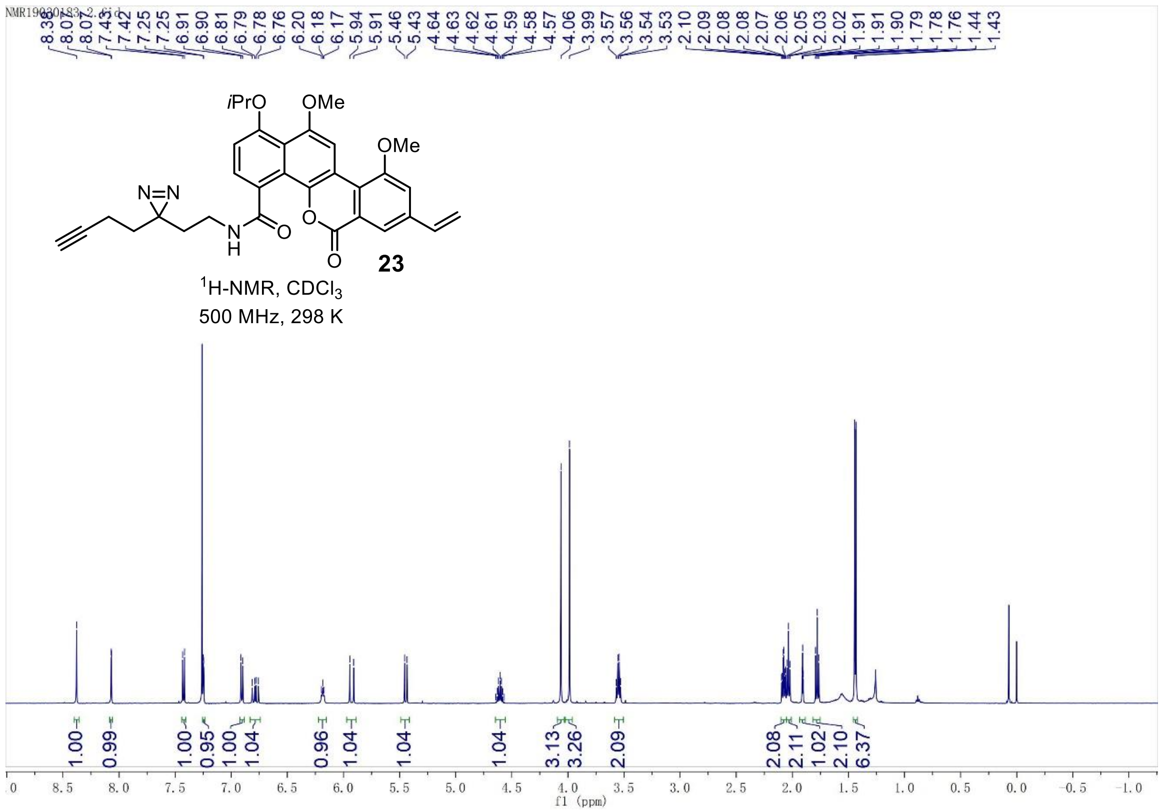
NMR19030183-2.fid
8.38 8.07 8.07 7.43 7.42 7.25 7.25 6.91 6.90 6.81 6.79 6.78 6.76 6.20 6.18 6.17 5.94 5.91 5.46 5.43 4.64 4.63 4.62 4.61 4.59 4.58 4.57 4.06 3.99 3.57 3.56 3.54 3.53 2.10 2.09 2.08 2.08 2.07 2.06 2.05 2.03 2.02 1.91 1.91 1.90 1.79 1.78 1.76 1.44 1.43
iPrO
OMe
OMe
N=N
N
H
O
O
O
23
^{1}H-NMR, $CDCl_3$
500 MHz, 298 K
1.00 0.99 1.00 0.95 1.00 1.04 0.96 1.04 1.04 1.04 3.13 3.26 2.09 2.08 2.11 1.02 2.10 6.37
f1 (ppm)

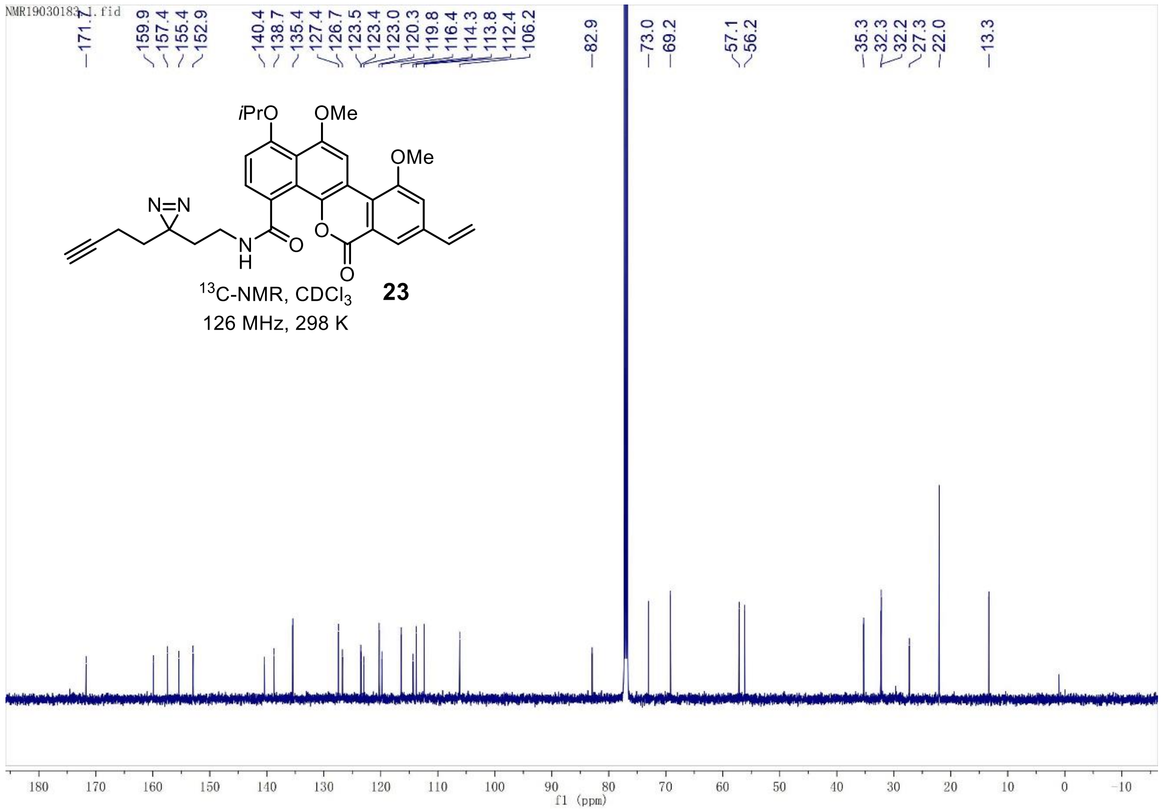
NMR19030183-1.fid
171.7 159.9 157.4 155.4 152.9 140.4 138.7 135.4 127.4 126.7 123.5 123.4 123.0 120.3 119.8 116.4 114.3 113.8 112.4 106.2 82.9 73.0 69.2 57.1 56.2 35.3 32.3 32.2 27.3 22.0 13.3
iPrO
OMe
OMe
N=N
N
H
O
O
O
^{13}C-NMR, $CDCl_3$
126 MHz, 298 K
23
f1 (ppm)

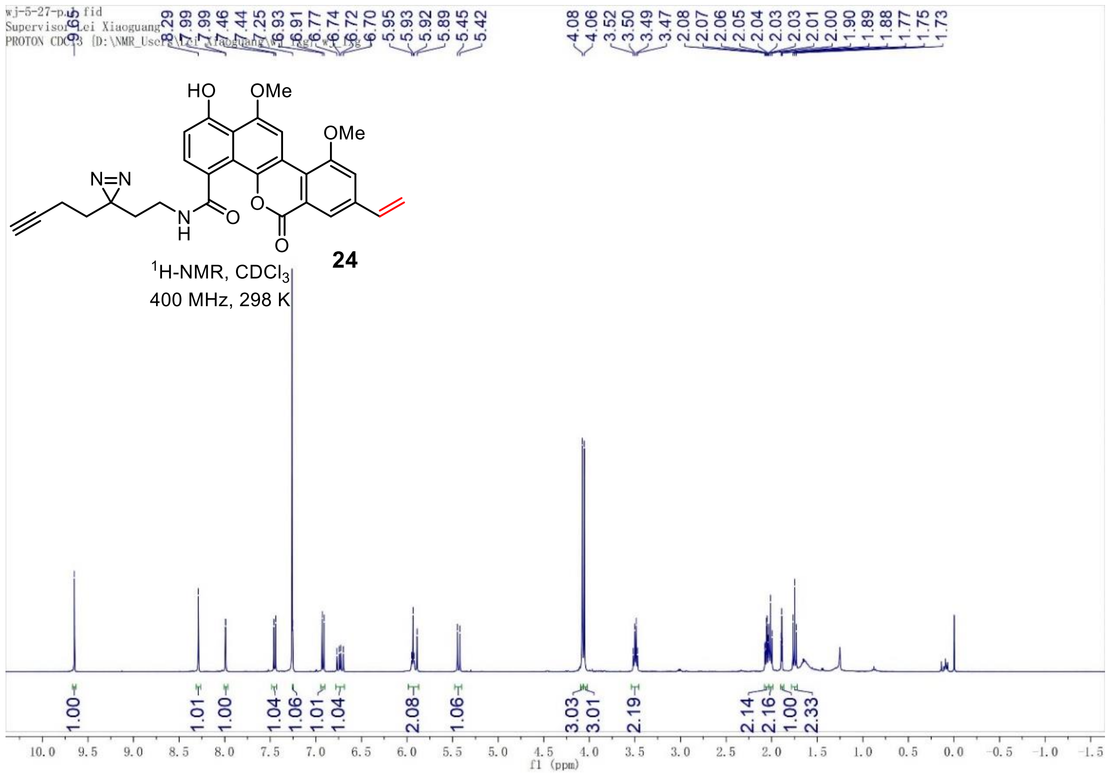

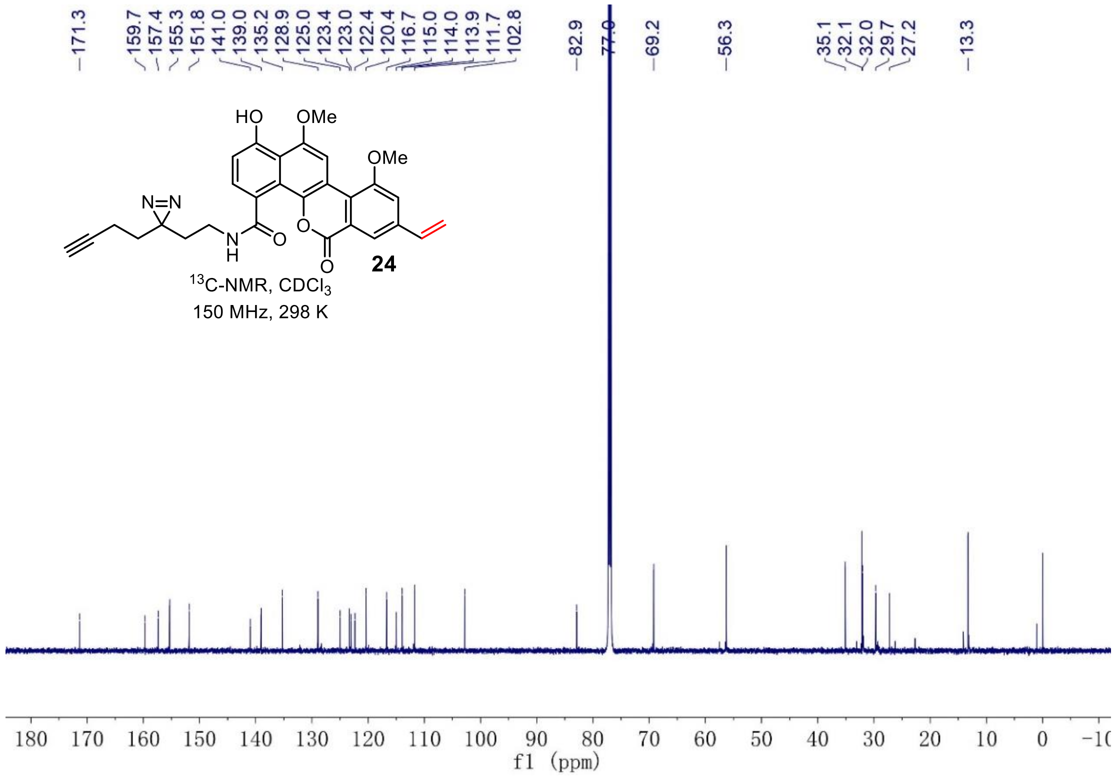

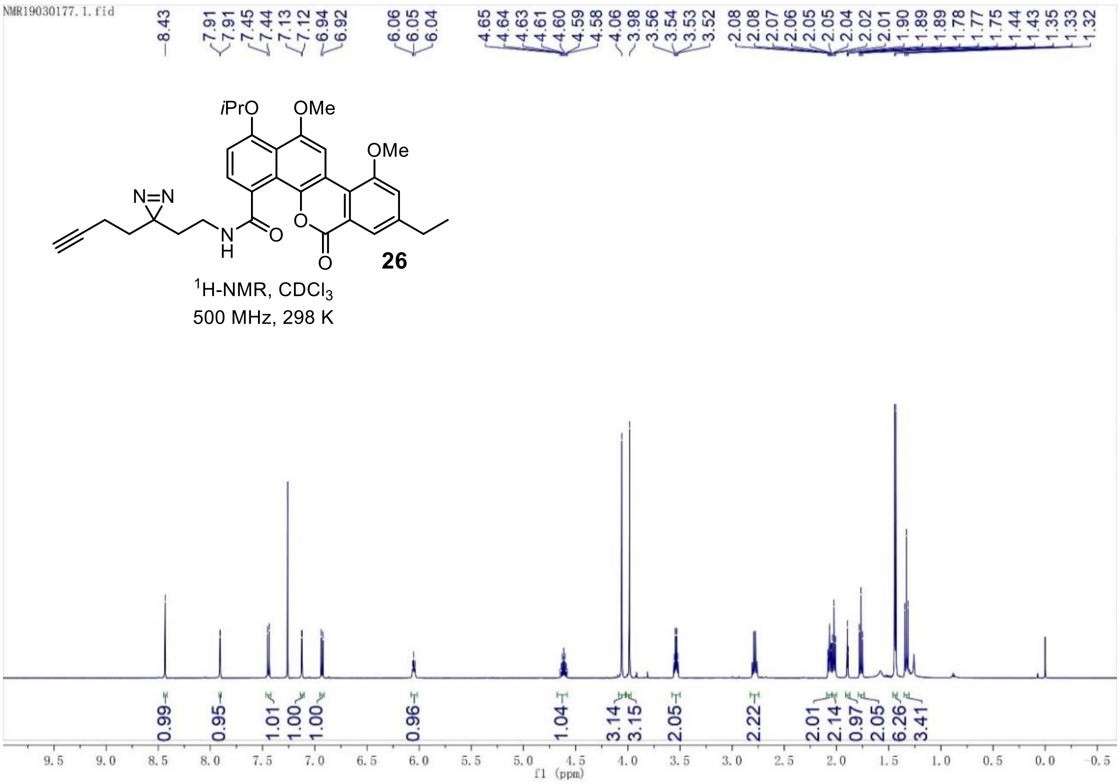
NMR19030177.1.fid
8.43
7.91
7.91
7.45
7.44
7.13
7.12
6.94
6.92
6.06
6.05
6.04
4.65
4.64
4.63
4.61
4.60
4.59
4.58
4.06
3.98
3.56
3.54
3.53
3.52
2.08
2.08
2.07
2.06
2.05
2.05
2.04
2.02
2.01
1.90
1.89
1.89
1.78
1.77
1.75
1.44
1.43
1.35
1.33
1.32
iPrO
OMe
OMe
N=N
N
H
O
O
O
26
^{1}H-NMR, $CDCl_3$
500 MHz, 298 K
0.99
0.95
1.01
1.00
1.00
0.96
1.04
3.14
3.15
2.05
2.22
2.01
2.14
0.97
2.05
6.26
3.41
9.5
9.0
8.5
8.0
7.5
7.0
6.5
6.0
5.5
5.0
4.5
4.0
3.5
3.0
2.5
2.0
1.5
1.0
0.5
0.0
-0.5
f1 (ppm)

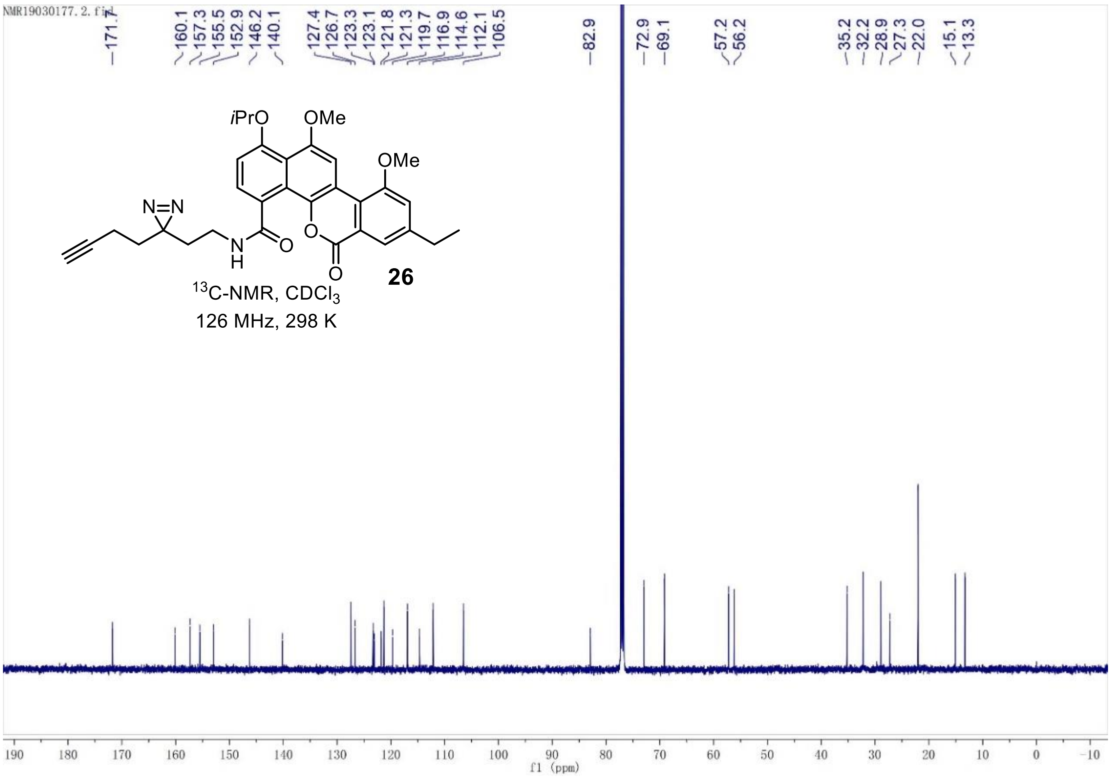
NMR19030177.2.fid
171.7
160.1
157.3
155.5
152.9
146.2
140.1
127.4
126.7
123.3
123.1
121.8
121.3
119.7
116.9
114.6
112.1
106.5
82.9
72.9
69.1
57.2
56.2
35.2
32.2
28.9
27.3
22.0
15.1
13.3
iPrO
OMe
OMe
N=N
N
H
O
O
O
26
^{13}C-NMR, $CDCl_3$
126 MHz, 298 K
190
180
170
160
150
140
130
120
110
100
90
80
70
60
50
40
30
20
10
0
-10
f1 (ppm)

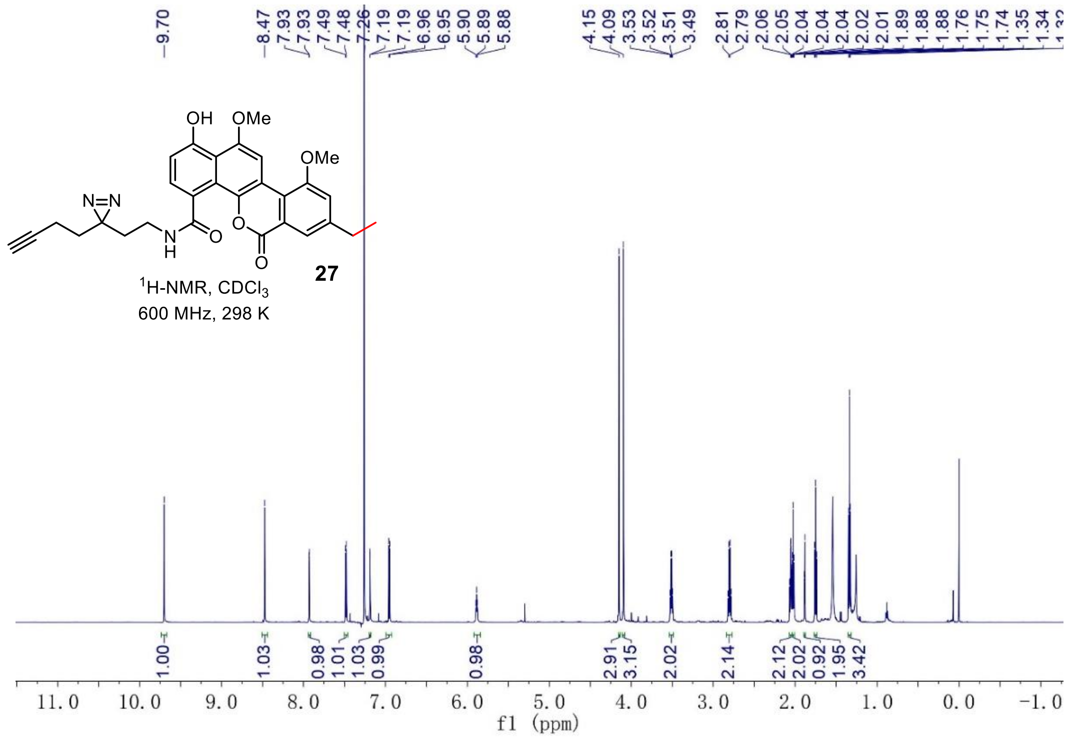

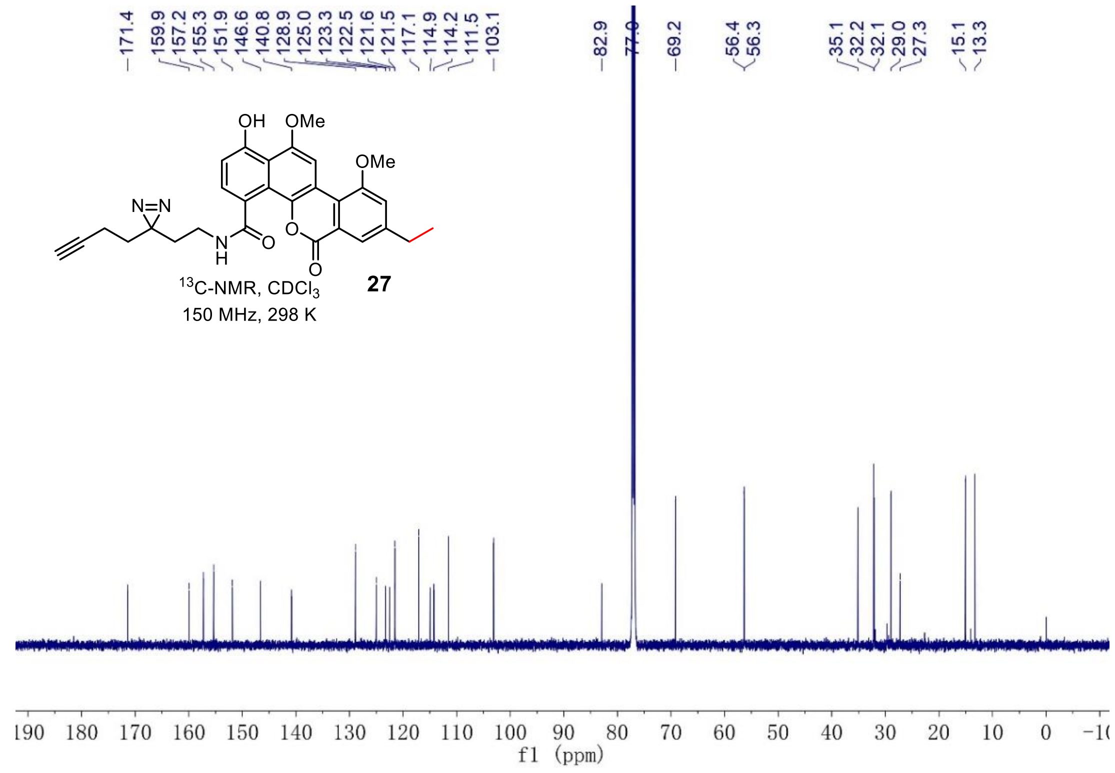

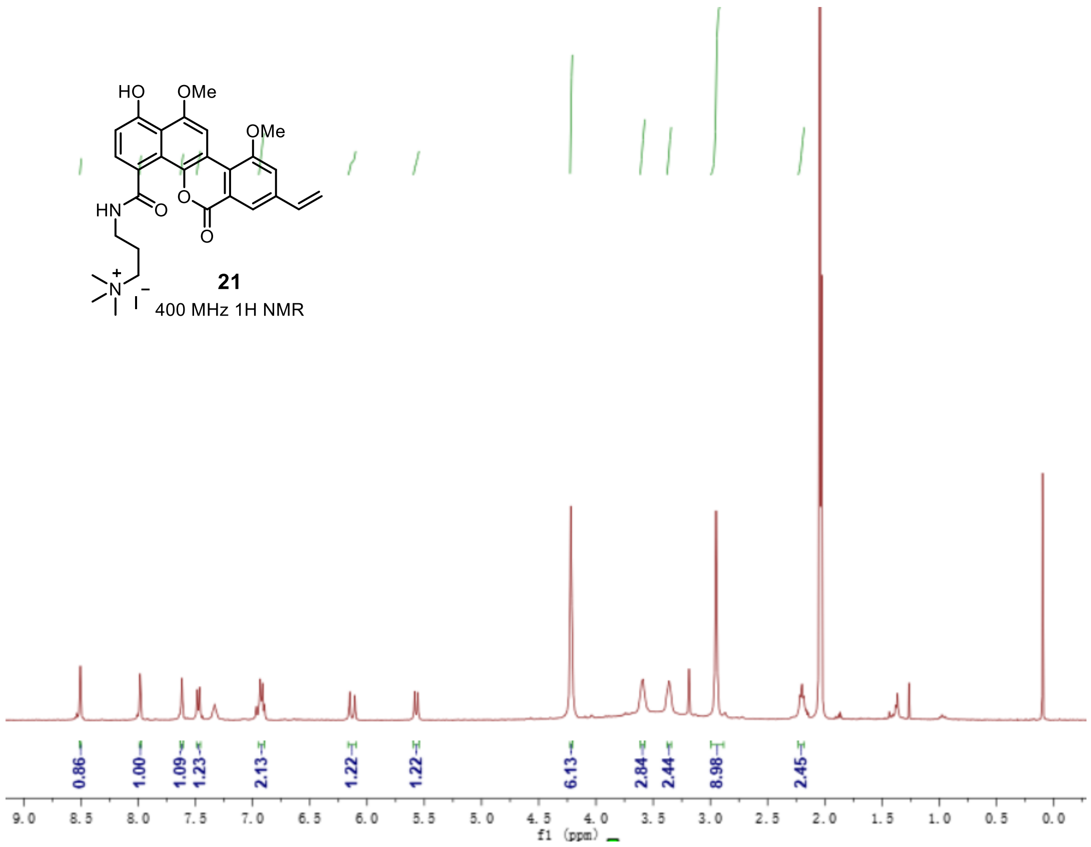

21

400 MHz 1H NMR

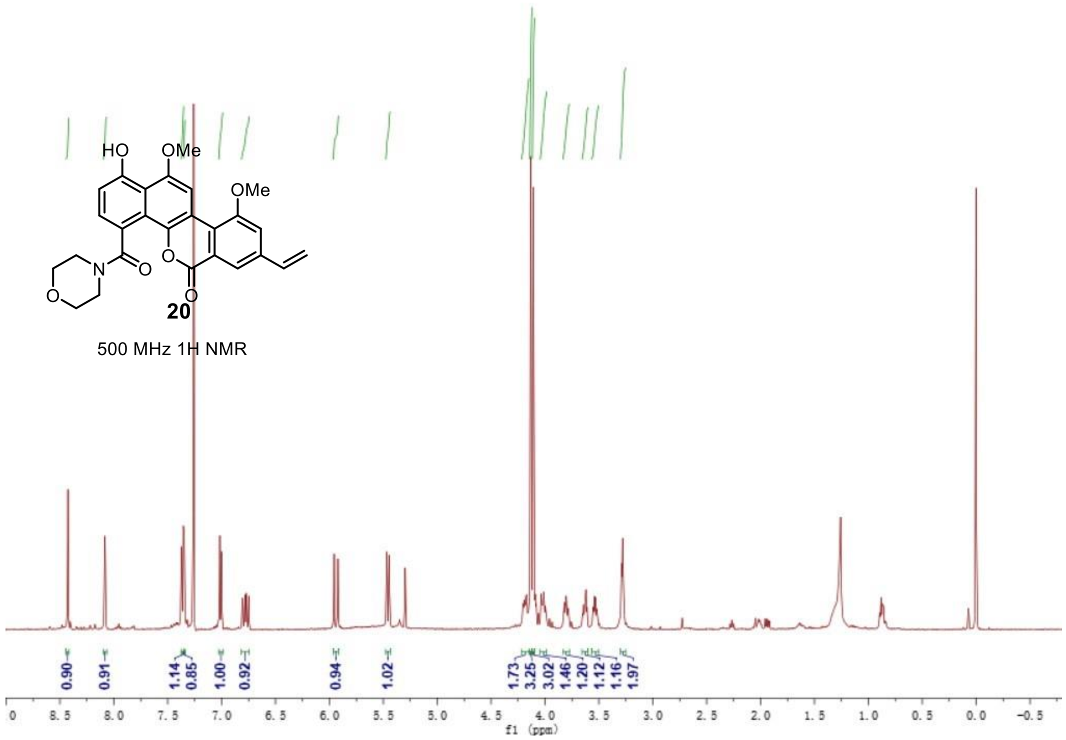

20

500 MHz 1H NMR

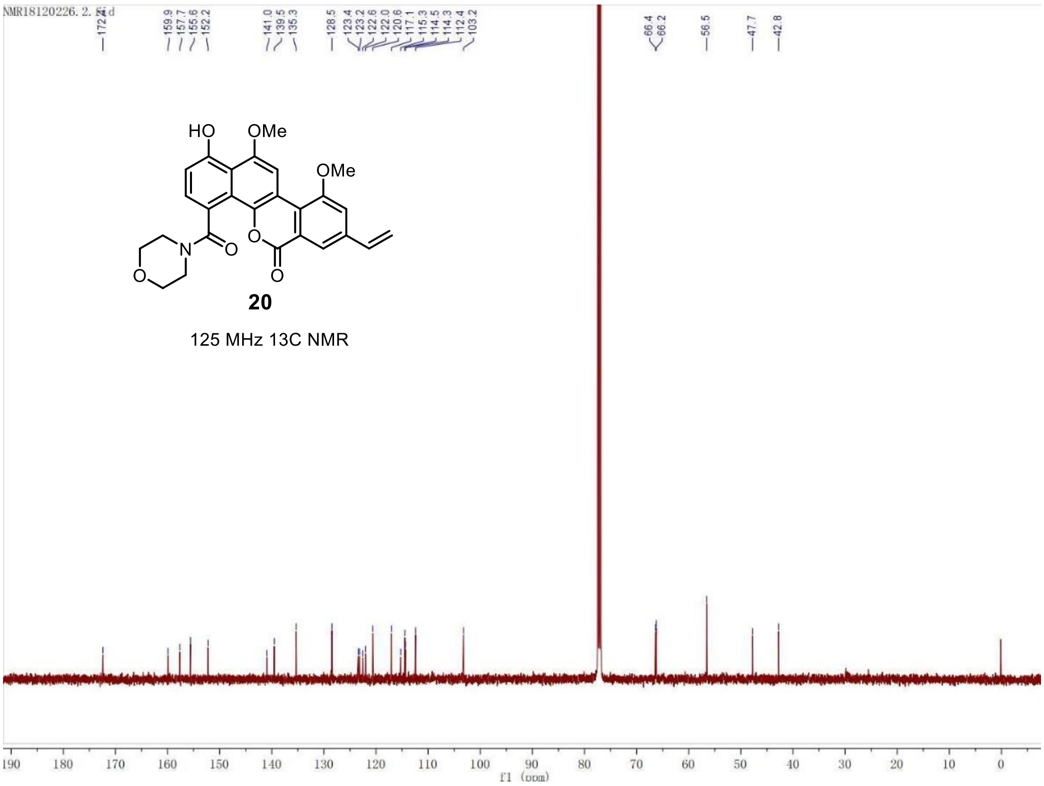

Reference:

1. Wu F., Zhang J., Song F., Wang S., Guo H., Wei Q., Dai H., Chen X., Xia X., Liu X., Zhang L., Yu J.Q., Lei X. Chrysomycin A Derivatives for the Treatment of Multi-Drug-Resistant Tuberculosis. *ACS Cent Sci.* **2020**, *6* (6), 928-938..
2. Gasparro F.P., Psoarlen-DNA Interactions: Thermodynamics and Photochemistry in "Psoralen-DNA Photobiology", *CRC Press, Inc., Boca Raton*, FL, **1988**.
3. Li, Z.; Hao, P.; Li, L.; Tan, C. Y.; Cheng, X.; Chen, G. Y.; Sze, S. K.; Shen, H. M.; Yao, S. Q. Design and synthesis of minimalist terminal alkyne-containing diazirine photo-crosslinkers and their incorporation into kinase inhibitors for cell- and tissue-based proteome profiling. *Angew. Chem., Int. Ed.* **2013**, *52* (33), 8551−8556.
